# Evolutionary Implications of Self-Assembling Cybernetic Materials with Collective Problem-Solving Intelligence at Multiple Scales

**DOI:** 10.3390/e26070532

**Published:** 2024-06-21

**Authors:** Benedikt Hartl, Sebastian Risi, Michael Levin

**Affiliations:** 1 Allen Discovery Center, Tufts University, Medford, MA 02155, USA; hartl.bene.research@gmail.com; 2 Institute for Theoretical Physics, Center for Computational Materials Science (CMS), TU Wien, 1040 Wien, Austria; 3 Digital Design, IT University of Copenhagen, 2300 Copenhagen, Denmark; sebr@itu.dk; 4 Wyss Institute for Biologically Inspired Engineering, Harvard University, Boston, MA 02115, USA

**Keywords:** evolution, multi-scale competency, artificial intelligence, swarm intelligence, cells, embryos, development, self-assembly

## Abstract

In recent years, the scientific community has increasingly recognized the complex multi-scale competency architecture (MCA) of biology, comprising nested layers of active homeostatic agents, each forming the self-orchestrated substrate for the layer above, and, in turn, relying on the structural and functional plasticity of the layer(s) below. The question of how natural selection could give rise to this MCA has been the focus of intense research. Here, we instead investigate the effects of such decision-making competencies of MCA agential components on the process of evolution itself, using in silico neuroevolution experiments of simulated, minimal developmental biology. We specifically model the process of morphogenesis with neural cellular automata (NCAs) and utilize an evolutionary algorithm to optimize the corresponding model parameters with the objective of collectively self-assembling a two-dimensional spatial target pattern (reliable morphogenesis). Furthermore, we systematically vary the accuracy with which the uni-cellular agents of an NCA can regulate their cell states (simulating stochastic processes and noise during development). This allows us to continuously scale the agents’ competency levels from a direct encoding scheme (no competency) to an MCA (with perfect reliability in cell decision executions). We demonstrate that an evolutionary process proceeds much more rapidly when evolving the functional parameters of an MCA compared to evolving the target pattern directly. Moreover, the evolved MCAs generalize well toward system parameter changes and even modified objective functions of the evolutionary process. Thus, the adaptive problem-solving competencies of the agential parts in our NCA-based in silico morphogenesis model strongly affect the evolutionary process, suggesting significant functional implications of the near-ubiquitous competency seen in living matter.

## 1. Introduction

Biological systems are organized in an exquisite architecture of layers, including molecular networks, organelles, cells, tissues, organs, organisms, swarms, and ecosystems. It is well recognized that life exhibits complexity at every scale. Increasingly realized, however, is the fact that those layers are not merely complex but actually active “agential matter”, which has agendas and competencies of its own [1,2]. Elsewhere, we have discussed examples of problem-solving in unconventional spaces, including transcriptional, physiological, metabolic, and anatomical space [3].

Especially interesting is the ability of these ubiquitous biological agents to deal with novel situations on the fly, which is not limited to brainy animals navigating 3D space but also occurs with respect to injury, mutations, and other kinds of external and internal perturbations (reviewed in [4]). One example of such problem-solving capabilities is the regenerative properties of some species that can regrow limbs, organs, or entire parts of their bodies when amputated, and—remarkably—stop when the precisely correct target morphology is complete [5,6,7]. This can be understood as cellular collectives navigating morphospace until the desired target shape—or the goal—is reached again. Other examples include the ability of scrambled tadpole faces to reorganize in novel ways to result in normal frog faces [8], and the normal shape and size of structures in amphibia despite drastic changes in cell number [9] and cell size [10], which are handled by exploiting different molecular mechanisms to reach correct target morphologies despite novel changes in internal components. Behavioral and morphological plasticity intersect in cases such as tadpoles made with eyes on their tails, which nevertheless can see and learn in visual assays without needing rounds of evolutionary adaptation [11].

The ability to navigate transcriptional and anatomical spaces, using perception–action loops and homeostatic setpoints, is now being increasingly targeted by biomedical and bioengineering efforts [12,13]. A fascinating body of work exists around the question of how neural and non-neural problem-solving capacities evolved, and how neuro-behavioral intelligence affects evolution [14,15,16,17,18,19,20,21,22,23,24,25,26,27,28,29,30,31]. However, we and others have previously suggested that somatic competency pre-dates neural intelligence [32,33,34], and has a bi-directional interaction with the evolutionary and developmental process [1,3,35]. Thus, here, we address the second half of the evolution–intelligence spiral: how are evolutionary processes affected by the competency of the material? Especially important is the inclusion of the middle layer between the genotype and phenotype. Mutation operates on genomes, and selection operates on phenotypic performance, but in most organisms, the connection between them is not linear or shallow—instead, developmental physiology provides a deep reservoir of dynamics that strongly alter the process. As a contribution to the study of evolvability and developmental mechanisms potentiating it [36,37,38,39,40,41,42,43,44,45,46,47,48,49,50,51,52,53], we established a virtual embryogeny [54] system focused on anatomical morphogenesis by cells. In this minimal model of morphogenesis, we were able to study the effects of different degrees of cellular competency on the evolutionary process.

**Figure 1:**
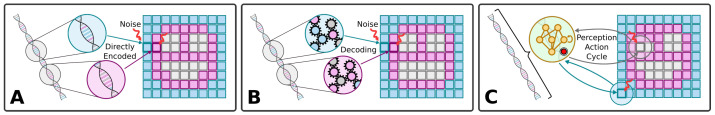
(**A**–**C**) Illustration of different ways of genetic encodings of a phenotype of, here, a two-dimensional smiley-face tissue composed of single cells. (**A**) Direct encoding: Each gene encodes a specific phenotypic trait, here, of each specific cell type of the tissue, colored blue, pink, and white. (**B**) Indirect encoding: A deterministic mapping between the genome and different phenotypic traits, here, again of each cell type (shown for completeness, but not investigated here due to reasons discussed in the Section 5). (**C**) Multi-scale competency architecture: Encoding of functional parameters of the uni-cellular agents which self-assemble a target pattern via successive local perception–action cycles [1] (as detailed in Figure 2A). In all three panels, we schematically illustrate, from left to right, the genome, the respective encoding mechanism, and the corresponding phenotype; colors indicate cell types, and arrows indicate the flow of information and environmental noise, affecting each cell during the developmental process.

The standard understanding of (Neo-Darwinian) evolution is schematized in Figure 1A: The *genome* of an organism encodes aspects of the organism’s cellular hardware, which together define the phenotypic traits. Given a competitive environment, natural selection then favors organisms with advantageous traits, and thus, on average, the corresponding genes tend to get passed on to the next generations more frequently. Random mutations may occur, consequently changing traits in the offspring phenotype. This affects the offspring’s reproductive success during the selection stage and, in that way, good traits prevail, and bad ones perish over time.

This view has been revised by Waddington [55,56], and more recent works [57,58,59,60,61,62,63,64,65,66], and has been the subject of vigorous debate [40,63,67,68,69,70,71,72] with respect to its capabilities for discovery, its optimal locus of control, and the degree to which various aspects are random (uncorrelated to the probability of future fitness improvements). Important open questions concern ways in which the properties of development—the layer between the mutated genotype and the selected phenotype—are evolved and in turn affect the evolutionary process [36,39,45,46,73,74,75,76,77,78]. Specifically, significant work has been conducted at the interface of evolution and learning—selectionist accounts of change and variational accounts of change respectively [30,61,62,66,79,80,81,82,83,84,85]. Significant progress has been made on the question of how evolution produces agents with behavioral competency in diverse problem spaces [17,86,87,88]. We have focused on a particular kind of competency—that of navigating anatomical morphospace [3,12,89,90]. More specifically, we here investigate in silico the evolutionary implications of the self-orchestrated process of morphogenesis, where local actions of single cells need to be aligned with a global policy of a multi-cellular collective to guide the formation of a large-scale tissue, in turn affecting the underlying evolutionary process. Work on developmental plasticity, chimeras, synthetic biobots, and the ability to overcome novel stressors has highlighted ways in which evolution seems to give rise to problem-solving machines, not fixed solutions to specific environments [91].

**Figure 2:**
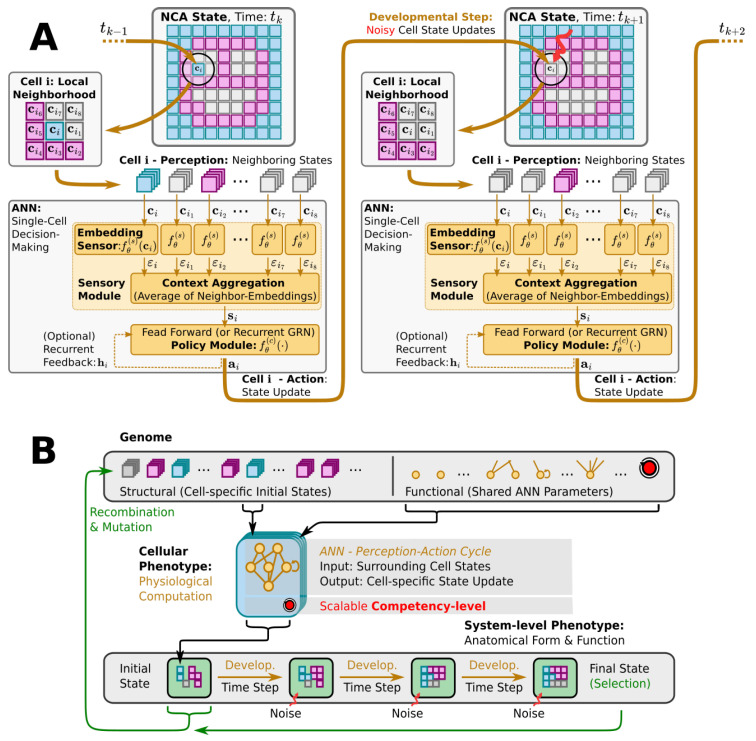
(**A**) Detailed information flow-chart of the perception–action cycle of a particular single cell agent, labeled *i*, in a neural cellular automaton (NCA)-based multi-scale competency architecture (cf. Figure 1C and Section 3.1): Starting from a multi-cellular phenotype configuration at time $t_{k}$ (left smiley-face panel), and following the thick orange arrows, each cell *i* perceives cell state information about its respective local neighborhood of the surrounding tissue (respectively labeled). This input is passed through an artificial neural network (ANN), substituting the internal decision-making machinery of a single cell, until an action output is proposed that induces a (noisy) cell state update in the next developmental step at time $t_{k+1}$ (details on labeled internal ANN operation and ANN architectures are introduced later in Section 3.1 and Appendix A). (**B**) Schematic illustration—following Ref. [1]—of the evolution of a morphogenesis process with a multi-scale competency architecture acting as the developmental layer between genotypes and phenotypes (see Section 3.1 and Section 3.2 for details): The genotype (top) encodes the structural (initial cell states) and functional parts (decision-making machinery) of a uni-cellular phenotype (center). The cell’s decision-making machinery is represented as a potentially recurrent ANN (yellow/orange graph) with an adjustable competency level (red knob). Through repeated local interactions (perception–action cycles; detailed in panel (**A**), the multi-cellular collective self-orchestrates the iterative process of morphogenesis and forms a final target pattern, i.e., a system-level phenotype after a fixed number of developmental steps (bottom left to right) while being subjected to noisy cell state updates at each step (red arrows). The evolutionary process solely selects at the level of the system-level phenotypes (labeled *Final State* at the bottom right). Based on a phenotypic fitness criterion, the corresponding genotypes, composed of the initial cell states (bottom left) and the functional ANN parameters (top right, are subject to evolutionary reproduction—recombination and mutation operations—to form the next generation of cellular phenotypes that successively “compute” the corresponding system-level phenotypes via morphogenesis, etc.

Thus, the problem-solving capacities of development, regeneration, and remodeling ensure that in many (perhaps most) kinds of organisms, the mapping from genotype to phenotype is not merely complex and indirect [92] (as schematized in Figure 1B) but actually enables evolution to search the space of behavior-shaping signals, not microstates, and exploit the modularity and triggers of complex downstream responses (cf. Figure 1C). We have previously argued that both evolution and human bioengineers face a range of unique problems and opportunities when dealing with the agential material of life—not passive or even just active matter but a substrate that has problem-solving competencies and agendas at many scales [93,94]. What selection sees is not the actual quality of the genome but the quality of the form and function of the flexible physiological “software” that runs on the genomically specified molecular hardware as schematically illustrated in Figure 2. This in turn suggests that the actual progress of evolution should be significantly impacted by the degree and kind of competency in the developmental architecture. Prior work has suggested a powerful feedback loop between the evolution of morphogenetic problem-solving and the effects of these competencies on the ability of evolutionary search to produce adaptive complexity [1,35,95]. Here, we construct and analyze a new model of evolving morphogenesis to study how different competency architectures within and among cells impact evolutionary metrics such as rate, robustness to noise, and transferability to new environmental challenges.

To quantitatively study the effects that different levels of competency of the decision-making centers in a multi-scale competency architecture have on the process of evolution, we here rely on tools from the research field of Artificial Life [96], which furthers computational and cybernetic models that mimic life-like behavior based on ideas taken from biology; a simple example is cellular automata (CAs) [97]. In such CAs, the (numerical) states of localized cells, organized on a discrete spatial grid, change in time via local update rules. Although typically rather simple “hardcoded” update rules are employed, CAs often display complex dynamics (cf. Conway’s *Game of Life* [98] or *Lenia* [99]) but are not known to exhibit homeostatic (closed-loop) activity. An extension of CAs, termed neural cellular automata (NCAs) [100], utilize artificial neural networks (ANNs) as more flexible trainable update rules, aiming to model the internal decision-making machinery of biological cells. Employing machine learning methods, such NCAs have been trained to perform self-orchestrated pattern formation (notably, of images from a single “seed” cell) [101] and even the co-evolution of a rigid robot’s morphology, and its controller has been demonstrated with such NCAs [102].

NCAs exhibit a striking resemblance to the genome-based multi-scale competency architecture of biological life [102] as illustrated in Figure 2: an organism’s entire building plan is encoded in its genome (corresponding to the NCA parameters), while its cells collectively run the self-orchestrated developmental program of morphogenesis (realized by the NCA layout and ANN architecture) via perception–action cycles at the uni-cellular level (cell state updates in the NCA, cf. Figure 2A). Starting from an initial cell state configuration of the NCA, the details of a virtual organism are then, step by step, “refined” in a collective self-organizing growth phase on the cellular level, and maintained against cell state errors later on in the virtual organism’s lifetime. Thus, a single NCA, once trained, guides the growth and integrity of a virtual organism’s tissue via intracellular information processing and intercellular communication, imitating in silico the multi-scale competency-based process of morphogenesis and morphostasis. (Notably, although an NCA update function—the cells’ ANN—is trainable in principle, current approaches pre-train (or evolve, as in our case) the ANN parameters to subsequently study the NCA behavior (such as simulated morphogenesis). Thus, an NCA’s self-orchestrated (developmental) program is defined by a particular set of ANN parameters rather than being acquired during the lifetime of the NCA. Here, we investigate the efficiency at which an evolutionary process arrives at satisfying parameters under various conditions.)

Here, we deploy a swarm of virtual uni-cellular agents on the spatial grid of an NCA. As illustrated in Figure 2A, each uni-cellular agent’s internal decision-making machinery is modeled by an ANN that allows each agent to independently perceive the cell states of its adjacent neighbors on the grid and propose cell state update actions to regulate its own cell state over time. The collective of cells thereby forms a spatial pattern or tissue of cell states on the NCA via local communication rules.

We utilize evolutionary algorithms (EAs) [103] as a simulated evolutionary process to optimize the parameters of such NCAs, so the uni-cellular agents evolve to collectively self-assemble a predefined target pattern of cell states in a fixed number of developmental steps; see Figure 2B for a flow-chart of the evolutionary process. We explicitly separate the NCA parameters into a structural and a functional part. The structural parameters describe the initial cell state, and the functional parameters the weights and biases of the ANN of each agent as illustrated by the “Genome” in Figure 2B. Both the structural and functional parts of the genome are compiled into a swarm of uni-cellular phenotypes on the grid of the NCA. Thus, starting from an initial cell state configuration, given by the structural part of the genome, the uni-cellular agents of the NCA run the developmental program of morphogenesis via successive perception–action cycles (see Figure 2A) to self-assemble in successive developmental steps a system-level phenotype, i.e., a two-dimensional pattern of cell states on the NCA. The deviation of these final cell state configurations from a desired target pattern—here, a Czech flag pattern or smiley-face pattern reminiscent of that of the amphibian craniofacial pre-pattern [104]—defines the phenotypic fitness score of a particular NCA realization. Based on an entire population of NCAs, and on the corresponding fitness scores, the EA successively samples the genomes of the next generation of NCAs, which, over time, evolve to reliably self-assemble the target pattern.

The conceptually simple process of cell state updates of NCAs and the ANN-based modeling of the uni-cellular decision-making allow us to interfere with (I) the reliability of the cell state update executions, and (II) with the computational capacity of the ANNs that guide each cell’s decision-making. To vary the former (I), we introduce a decision-making probability $P_{D}$ that specifies the probability at which a proposed update of each individual cell is executed in the environment (or omitted otherwise). Thus, by tuning the decision-making probability from $P_{D}=0$ to $P_{D}=1$, we can continuously vary the behavior of the NCA from a direct-encoding scheme without competency to a multi-scale competency architecture with perfect reliability in cell state update executions.

To systematically vary the computational capacity of the involved ANNs (II), we introduce independent copies of a particular sub-module of the uni-cellular agents’ ANNs, i.e., of the policy module illustrated in Figure 2A (see Section 3.1 and Section 4.2 and Appendix A for details on the ANN architectures). This increases the number of evolvable parameters of the ANNs, which are responsible for performing the same operation, namely, interpreting the cell’s local environment and proposing a cell state update action. Thus, by taking the average output of all redundant policy modules of a single agent, a cell’s decision-making can be biased by the several redundant paths through which signals are transmitted in the ANN, inspired by error-correcting codes [105,106,107]. We explicitly define a redundancy number *R* that specifies how many redundant copies of the policy module are used in the ANNs of the cells of the NCA.

The decision-making probability (I) and the redundancy number (II) represent two levels of competency in our system (schematically illustrated by the red arrowin Figure 1C and Figure 2B), which we can scale continuously (I) or discretely (II) to systematically tune the behavior of an NCA. Throughout the manuscript, we refer to these two parameters as “competency levels”, but we would like to stress that many more options would have been possible to vary the competency in our system. For instance, the particular ANN architecture can have large effects on the competency of the uni-cellular agents; a systematic investigation thereof is out of the scope of this work. Here, we utilize two particular ANN architectures, one based on a Feedforward (FF) and one based on a recurrent ANN architecture [108] that is inspired by gene regulatory networks (GRNs) [109], which we thus term recurrent gene regulatory network (RGRN), see Appendix A for details.

To study the effects of different competency levels of the decision-making centers in a multi-scale competency architecture on the underlying evolutionary process of a morphogenesis task, we systematically vary in large-scale simulations the decision-making probability (I) and the redundancy number (II) of NCAs with FF and RGRN ANN architectures. Furthermore, we expose the corresponding NCAs to different noise conditions during cell state updates (III) and perform several statistically independent evolutionary searches at each parameter combination (I–III) to investigate the performance of the evolutionary process of finding solutions to such noisy pattern formation tasks.

The manuscript is organized as follows: In Section 3, we describe the numerical and computational methods applied herein. More specifically, we introduce NCAs in Section 3.1, and describe the neuroevolution approach used to optimize the NCAs ANN parameters based on ideas of evolution and natural selection via EAs in Section 3.2. We specify the particular morphogenetic problem we primarily focused on—the 8×8 Czech flag task—in Section 4.1, and compare in Section 4.2 the efficiency of evolving the target pattern via a direct encoding scheme and a multi-scale competency architecture. In Section 4.3, we functionally define and systematically vary the different tunable competency levels in our system to illustrate the evolutionary implications of utilizing a multi-scale competency architecture rather than a direct encoding scheme for morphogenesis tasks. We then study the effects of allowing the evolutionary process to afford competency as a gene during optimization in Section 4.4, and eventually investigate our multi-scale competency approach for robustness and generalizability regarding system parameter changes in Section 4.5, and for transferability to modified target patterns in Section 4.6. We conclude in Section 5, and attach an appendix.

## 2. General Summary

Biological systems are composed of layers of organization, each level providing the foundation for the next higher level of abstraction: membranes, DNA, and proteins form cells, which then collectively organize into tissue and, in further hierarchical steps, into tissues, organs, bodies, swarms, ecosystems, etc. Each of these layers has a degree of ability to adapt in real-time to new conditions to establish and maintain specific outcomes in terms of physiological, metabolic, transcriptional, and anatomical spaces. In other words, evolution works with material that is not passive matter but rather has a degree of competency—an agential material that forms the layer between the genotype and the phenotype. Many scientific studies have been dedicated to investigating how evolution gives rise to such intriguing problem-solving machines we call organisms. In this study, we ask the reverse question: what is it like to evolve over such a material vs. one that passively maps genotypes into the form and function that selection operates over—how does it affect the process of evolution itself? We test this in silico by utilizing evolutionary algorithms to adapt the behavior of a swarm of virtual uni-cellular agents in large-scale simulations of virtual embryos. In our minimal model, the cells collectively self-assemble a predefined target tissue on a neural cellular automaton. We find that competency at the cellular level of our multi-scale model system strongly affects the resulting evolutionary process, as well as the generalizability, evolvability, and transferability of the evolved solutions, suggesting the profound evolutionary implications of the highly intricate multi-scale competency architecture of biological life.

## 3. Methods

### 3.1. Neural Cellular Automaton: A Multi-Agent Model for Morphogenesis

*Cellular automata* (CAs) have been introduced by von Neumann to study self-replicating machines [97] and are simple models for *Artificial Life* [96]. In CAs, a discrete spatial grid of cells is maintained over time, each cell *i* being attributed a binary, integer, real, or even vector-valued state $c_{i}\left(t_{k}\right)$ at each step in time $t_{k}$. The cell states evolve over time via local updated rules $c_{i}\left(t_{k+1}\right)=f_{u}\left(N_{i}\left(t_{k}\right)\right)$ as a function of its own $c_{i}\left(t_{k}\right)$ and the numerical states $c_{i_{\nu }}\left(t_{k}\right)$ of its $i_{\nu =1,\cdots ,N}$ neighboring cells on the grid that we collect in the matrix $N_{i}\left(t_{k}\right)=\left(c_{i_{0}}\left(t_{k}\right),\cdots ,c_{i_{N}}\left(t_{k}\right)\right)$ with $i_{0}\equiv i$. Although typically rather simple “hardcoded” (i.e., predefined) update rules $f_{u}\left(\cdot \right)$ are employed, CAs often display complex dynamics and can even be utilized for universal computation (cf. Conway’s *Game of Life* [98] or Wolfram’s *rule 110* [110,111]).

*Neural cellular automata* (NCAs) [100] extend CAs by replacing the local update rule with more flexible [112] artificial neural networks (ANNs) $f_{u}\left(\cdot \right)\to f_{\theta }\left(\cdot \right)$, where θ denotes the set of trainable parameters of the ANN (see Appendix A for details). Employing *Machine Learning*, such NCAs have been trained to perform self-orchestrated pattern formation [101] (notably, of RGB images from a single “seed” pixel) and even the co-evolution of a rigid robot’s morphology, and its controller has been demonstrated recently with NCAs in silico [102]. Such self-orchestrated pattern formation is reminiscent of the self-regulated development of a biological organism, from a single fertilized egg cell to a complex anatomical form. Thus, NCAs have been proposed as toy models for morphogenesis [101].

An NCA basically represents a grid of cells that are equipped with identical ANNs, each perceiving the numerical cell states of its host’s local environment, $N_{i}\left(t_{k}\right)$, and proposing actions, $a_{i}\left(t_{k}\right)=f_{\theta }\left(N_{i}\left(t_{k}\right)\right)$, to regulate its own cell state

(1) $$c_{i}\left(t_{k+1}\right)=c_{i}\left(t_{k}\right)+a_{i}\left(t_{k}\right)+\xi_{c},$$

and, in turn, the cell states of its neighbors—where we also account for potential noise $\xi_{c}$ in the environment during the process of morphogenesis. Thus, each cellular agent can only perceive the numerical states of its direct neighbors $N_{i}\left(t_{k}\right)$ at an instant of time $t_{k}$ and, in turn, communicate with these neighbors via cell state updates $c_{i}\left(t_{k+1}\right)$, following a policy $\pi \left(N_{i}\left(t_{k}\right)\right)\approx f_{\theta }\left(N_{i}\left(t_{k}\right)\right)$ that is approximated by an ANN with parameters θ. Through the lens of *Reinforcement Learning* [113], an NCA can thus be understood as a trainable, locally communicating multi-agent system that can be utilized such that the collective of cells achieves a target system-level outcome (see Appendix B for details).

In contrast to previous contributions of in silico morphogenesis experiments in NCAs [101], we here do not use standard convolutional filters in our ANN architectures but utilize permutation-invariant ANNs with respect to a cell’s neighbors, $N_{i}\left(t_{k}\right)$ (see Figure 2A for an illustration). Inspired by Ref. [114], this is achieved by partitioning a cell’s ANN into (i) a sensory part $f_{\theta }^{\left(s\right)}\left(\cdot \right)$, preprocessing its own, and the state of each neighboring cell separately into a respective sensor embedding $\varepsilon_{i_{\nu }}\left(t_{k}\right)=f_{\theta }^{\left(s\right)}\left(c_{i_{\nu }}\left(t_{k}\right)\right)\in R^{s}$, for $i_{\nu =0,\cdots ,N}$. These neighbor-wise sensor embeddings are (ii) averaged into a cell’s context vector $s_{i}\left(t_{k}\right)=\left[\frac{1}{N+1}\sum_{\nu =0}^{N}\varepsilon_{i_{\nu }}\left(t_{k}\right)\right]\in R^{s}$ of fixed size *s*, which is then used as the input of (iii) a controller ANN $f_{\theta }^{\left(c\right)}\left(\cdot \right)$, potentially with recurrent feedback connections, that eventually outputs the cell’s action $a_{i}\left(t_{k}\right)=f_{\theta }^{\left(c\right)}\left(s_{i}\left(t_{k}\right)\right)$; for details we refer to Appendix A.

Due to the mean aggregation of a cell’s sensory embedding, each cell completely loses its ability to spatially distinguish between neighboring (and even its own) state inputs and thus fully integrates into the tissue locally. We would like to stress the close relation of our approach to the concept of breaking down the computational boundaries of a cell’s “*Self*” via *forgetting* [93] and to the scaling of goals from a single agent’s to a system-level objective [95].

To model the developmental process of morphogenesis, we here employ NCAs on a two-dimensional $N_{x}\times N_{y}$ square grid with the objective that all cells of the grid assume their correct, predefined target cell type ${\hat{g}}_{i}$ after a fixed number of $t_{D}$ developmental time steps, starting from an initial cell state configuration $c_{i}\left(0\right)$. We attribute a number of $N_{G}$ elements $g_{i}\left(t_{k}\right)\in R^{N_{G}}$ of the $N_{C}$-dimensional cell state $c_{i}\left(t_{k}\right)\in R^{N_{C}}$ of an NCA as indicators for expressing one of $1,\cdots ,N_{G}$ discrete cell types such that $c_{i}\left(t_{k}\right)=g_{i}\left(t_{k}\right)\cup h_{i}\left(t_{k}\right)$; the remaining $N_{H}=\left(N_{C}-N_{G}\right)$ elements of the cell state represent hidden states $h_{i}\left(t_{k}\right)\in R^{N_{H}}$ of a cell that can be utilized by the NCA for intercellular communication. We explicitly define each cell’s type $g_{i}\left(t_{k}\right)$ as the argument (i.e., the index) of the maximum element of the $N_{G}$-dimensional indicator vector $g_{i}\left(t_{k}\right)$:

(2) $$g_{i}\left(t_{k}\right)=\underset{g\in R^{N_{G}}}{\arg\max}\left(g_{i}\left(t_{k}\right)\right).$$

Training an NCA to assemble a predefined target pattern (realized by a set of $N_{j}=N_{x}\times N_{y}$ target cell types $\left\{{\hat{g}}_{1},\cdots ,{\hat{g}}_{N_{j}}\right\}$ for the entire grid) thus boils down to finding a suitable set of NCA parameters (cf. “Genotype” in Figure 2B) that minimizes the deviation of each cell *i*’s type $g_{i}\left(t_{D}\right)$ from ${\hat{g}}_{i}$ after $t_{D}$ developmental time steps, i.e., after the developmental stage of the virtual organism (cf. “System-level Phenotype” in Figure 2B, from left to right, and details below). Here, we are interested in the *evolutionary implications of biologically inspired multi-scale competency architectures*, the latter being modeled by our morphogenetic NCA implementation. We thus introduce in Section 3.2, and utilize in Section 4, evolutionary algorithms to evolve suitable sets of NCA parameters that maximize the fitness score based on comparing the “final” cell types of the NCA, $g_{i}\left(t_{D}\right)$, with the predefined target cell types ${\hat{g}}_{i}$.

### 3.2. Neuroevolution of NCAs: An Evolutionary Algorithm Approach to Morphogenesis

*Evolutionary algorithms* (EAs) are heuristic optimization algorithms that maintain and optimize a set, i.e., a population $X=\{x_{1},\cdots ,x_{N_{P}}\}$ of parameters $x_{j}\in R^{X}$, also termed individuals, over successive generations to maximize an objective function, or a fitness score $r\left(x_{j}\right):R^{X}\to R$. Inspired by the ideas of natural selection and the DNA-based reproduction machinery of biological life, EAs (i) predominantly select the high-fitness individuals of a given population for reproduction, and utilize (ii) crossover and (iii) mutation operations to generate new offspring by (ii) merging the genomic material of two high-quality individuals from the current population $x_{o}=x_{j}⨁x_{k}$, and (iii) occasionally mutating the offspring genomes $x_{o}\to x_{o}+\xi_{x}$ by adding (typically Gaussian) noise to the parameters; the ⨁ symbol indicates a genuine merging operation of two genomes, which may depend on the particular EA implementation. In that way, a population X of individuals is guided towards high-fitness regions in the parameter space $R^{X}$, typically over many generations of successive selection and reproduction cycles (i)–(iii).

In contrast to biological life, many use cases of EAs do not require a distinction between individuals in the parameter space, i.e., *genotypes* $x_{j}$, and the corresponding organisms in their natural environment, i.e., *phenotypes*, $p_{j}$: while the genetic crossover and mutation operations of biological reproduction rely on bio-molecular mechanisms at the level of RNA and DNA, i.e., are performed in the genotype space, selection typically happens at the much more abstract level of an organism’s natural environment, i.e., in the phenotype space. Carrying this through computationally can be resource-demanding, depending on the complexity of a simulated environment. Nevertheless, to address the asymmetry between genotypes and phenotypes in multi-scale competency architectures, it is essential to evaluate the fitness score of the EA in the phenotype space instead of the genotype space $r\left(x_{j}\right)\to r\left(p_{j}\right)$.

We explicitly separate the genotype and phenotype representations of individuals by introducing a biologically inspired *developmental layer* [1] in between genotypes and phenotypes $x_{j}\overset{Dev.}{\underset{Layer}{\to }}p_{j}$ as illustrated in Figure 2. More precisely, we follow Section 3.1 and model the developmental process of morphogenesis in silico by utilizing NCAs: we treat an NCA *j*’s parameters, such as the set of $i=1,\cdots ,N_{j}$ initial cell states $x_{j}^{\left(S\right)}={\left\{c_{i}\left(0\right)\right\}}_{j}$ and the corresponding ANN parameters $x_{j}^{\left(F\right)}=\theta_{j}$, as the (virtual) organism’s genome,

(3) $$x_{j}=x_{j}^{\left(S\right)}\cup x_{j}^{\left(F\right)}=\left({\left\{c_{i}\left(0\right)\right\}}_{j},\theta_{j}\right),$$

explicitly partitioning the genome into a structural (S) and a functional (F) part, as indicated by the superscripts. We then perform a fixed number of $t_{D}$ developmental steps employing Equation (1) and interpret the corresponding set of “final” cell types ${\left\{g_{i}\left(t_{D}\right)\right\}}_{j}$ of the entire NCA, cf. Equation (2), as the mature phenotype,

(4) $$p_{j}={\left\{g_{i}\left(t_{D}\right)\right\}}_{j},$$

representing a two-dimensional tissue of cells.

In an effort to evolve the parameters $x_{j}$ of an NCA *j* to achieve the morphogenesis of a two-dimensional spatial pattern of cell types $p_{j}$ that resembles a pattern of predefined target cell types $\left\{{\hat{g}}_{1},\cdots ,{\hat{g}}_{N_{j}}\right\}$ of a total of $N_{j}$ cells on an $N_{x}\times N_{y}$ square grid (see Section 3.1), we define the phenotype-based fitness score $r(p_{j})$ as

(5) $$r\left(p_{j}\right)=\left(2n_{j}^{\left(G\right)}-N_{j}\right)+r_{T}\;n_{j}^{\left(T\right)}-r_{S}\;n_{j}^{\left(S\right)},$$

where (i) $n_{j}^{\left(G\right)}$ is the number of correctly assumed cell types $g_{i}\left(t_{D}\right)={\hat{g}}_{i}$ after $t_{D}$ developmental steps, (ii) $n_{j}^{\left(T\right)}$ is the number of time steps at which the entire target cell type pattern is correctly assumed, i.e., whenever $g_{i}\left(t_{k}\leq t_{D}\right)={\hat{g}}_{i}$ for all *i*, and (iii) $n_{j}^{\left(S\right)}$ is the number of successive time steps $t_{s}$ and $t_{s+1}\leq t_{D}$, where all cell types stagnate, i.e., where $g_{i}\left(t_{s+1}\right)=g_{i}\left(t_{s}\right)$ for all *i*. With Equation (5), we thus reward the entire NCA *j* by counting all correctly assumed cell types after $t_{D}$ developmental steps (while discounting all incorrect cell types $g_{i}\left(t_{D}\right)\neq {\hat{g}}_{i}$), we reward maintaining the target pattern over time with a factor of $r_{T}$, and discount a stagnation of a suboptimal pattern over time by a factor of $r_{S}$. We consider the problem solved if a final fitness score of $N_{j}=N_{x}\times N_{y}$ is reached. Notably, there is no explicit fitness or reward feedback at the level of the uni-cellular agents in our system; the fitness score is solely used as the selection criterion for sampling the next evolutionary generations, so the cellular collective needs to evolve an intrinsic signaling mechanism to successfully perform the requested morphogenesis task.

The here proposed setting of genotypes $x_{j}$, corresponding phenotypes $p_{j}$, and associated fitness scores $r(p_{j})$, given by Equations (3)–(5), respectively, can be used in combination with any black-box evolutionary or genetic algorithm. We rely on the well-established *Covariance Matrix Adaptation Evolutionary Strategy* (CMA-ES) [103] to simultaneously evolve the set of initial cell state configurations (i.e., structural genes, $x_{j}^{\left(S\right)}$) and the set of corresponding ANN parameters of an NCA (i.e., functional genes, $x_{j}^{\left(F\right)}$) with the objective of the purely self-orchestrated formation of two-dimensional spatial tissue as illustrated by Figure 2A and described by Equation (1).

## 4. Results

### 4.1. The System: An Agential Substrate Evolves to Self-Assemble the Czech Flag

Evolution works with an active rather than a passive substrate, i.e., with biological cells with agendas of their own [1]. Thus, at every stage of development during morphogenesis, collective decisions are made at vastly different length- and time scales within an organism, guiding the formation of the mature phenotype. We aim to model exactly this process via the neural cellular automata (NCAs) described in Section 3.1 and employ evolutionary algorithms (EAs) to evolve the parameters of such NCAs so the latter perform well on a target morphogenesis task, see Section 3.2. Without loss of generality, we consider an $N_{x}\times N_{y}=8\times 8$ Czech flag pattern (as a more complex version of the classic French flag problem of morphogenesis [115,116]) as the target pattern for our in silico morphogenesis experiments, with a fixed number of $N_{j}=64$ cells in total, $N_{G}=3$ distinct cell types (colored blue, white, and red, respectively) and $N_{H}=1$ hidden state, which renders the dimension of the NCA cell state $N_{C}=4$. We use a square grid of cells with N=8 neighbors per cell and with fixed boundary conditions (see Appendix C for details).

Starting from a genotype $x_{j}$ defined in Equation (3), we perform a number of $t_{D}=25$ developmental steps per morphogenesis experiment to “grow” a phenotype $p_{j}$, described by Equation (4), based on which the fitness score $r(p_{j})$ is evaluated following Equation (5) (see Figure 2B for an illustration of this process). During this entire process, we limit the magnitude of the numerical cell state values $c_{i}\left(t_{k}\right)$ at all time steps $t_{k}$ to the interval $l_{c}=\left[-3,3\right]$, and, analogously, limit the magnitude of the proposed actions $a_{i}\left(t\right)$ of each uni-cellular agent to the interval $l_{a}=\left[-1,1\right]$. This is achieved by clipping the numerical values of $c_{i}\left(t_{k+1}\right)$ after a cell state update described by Equation (1), and the ANN outputs $a_{i}\left(t\right)$ to the respective limits $l_{c}$ and $l_{a}$. The noise level $\xi_{c}$ defined in Equation (1) is counted in units of the action limits $\max(l_{a})$ and is thus sampled from a Gaussian distribution with zero mean and standard deviation $\xi_{c}$ independently for each of the $N_{C}=4$ cell state elements, thus affecting the cell state updates during development; the actual numerical values for the hyperparameters above turned out to be well suited for the problem at hand, especially to reasonably compare and discuss simulation results for the means of this contribution but are not crucial for the more general aspects of the evolutionary implications of multi-scale intelligence discussed here.

To study the effects of different types of decision-making machinery within a cell, we utilize two different architectures for the NCA artificial neural networks (ANNs), a Feedforward (FF) and a recurrent ANN inspired by gene regulatory networks [117,118,119,120] (RGRNs). (The terminology FF and RGRN stems from the respective agents’ *Feedforward* and *Recurrent Gene Regulatory Network* ANN controller layers (see Appendix A for details)). Notably, the RGRN-agent architecture augments cells with an internal memory that is independent of their states in the NCA and thus can not be accessed by the cells’ neighbors. To balance the length of the structural genome $x^{\left(S\right)}$ and functional genome $x^{\left(F\right)}$ defined in Equation (3), the two ANN architectures, FF and RGRN, are chosen such that the number of parameters $N_{\mathrm{FF}}=192$ and $N_{\mathrm{RGRN}}=164$ is roughly the same as the number of initial cell states $N_{j}\times N_{C}=64\times 4=256$. Thus, the ANNs utilized here—and detailed in Table A1 of Appendix A—are tiny compared to Ref. [101].

For each experiment of evolving the parameters of an NCA, i.e., for each independent run of the EA, we typically utilize a population X of $N_{P}=96$ individuals and a maximum number of $N_{M}=2000$ generations. As the EAs ultimate fitness criterion, we consider the average $F_{j}={\langle r\left(p_{j}\right)\rangle }_{N_{E}}$ of $N_{E}=8$ statistically independent fitness scores $r(p_{j})$ of corresponding morphogenesis simulations starting from an individual *j*’s genotype $x_{j}$ and resulting in a corresponding phenotype $p_{j}$ after $t_{D}$ developmental steps; the developmental program described via Equation (1) is imperfect due to the developmental noise applied to the cell state updates and can thus lead to different, noise-induced phenotypic realizations. Typical values used here for the corresponding reward factors defined in Equation (5) are $r_{T}=0.25$ and $r_{S}=0.5$. We consider the problem solved if a fitness of $F_{j}=\max\left(n_{j}^{\left(C\right)}\right)=N_{j}=64$ is reached, but since we reward individuals to maintain the target pattern over time (via $r_{T}$), the maximum possible fitness score after $t_{D}$ developmental time steps is $\max(r_{j}\left(p_{j}\right))=70.25$ in this example. Further details about the hyper-parameters of the EA and afforded computational resources can be found in Appendix D.

### 4.2. Direct vs. Multi-Scale Encoding: Cellular Competencies Affect System Level Evolvability

We aim in this contribution to investigate the evolutionary implications of biologically inspired multi-scale competency architectures [1,94]. Thus, we compare two qualitatively different evolutionary processes, both with the objective of morphogenetic pattern formation but whose genomes either (i) directly encode the phenotypic features of a two-dimensional target pattern (cf. Figure 1A), or (ii) encode the cellular competencies of a multi-scale architecture that gives rise to the same phenotypic features (cf. Figure 1C). Notably, different definitions of direct and indirect encodings in multi-agent systems have been used in the literature [54]. Here, we specifically distinguish between structural parameters $x_{j}^{\left(S\right)}={\left\{c_{i}\left(0\right)\right\}}_{j}$ in the search space that directly encode features of the phenotype, i.e., specific initial cell types $g_{i}\left(0\right)\approx {\hat{g}}_{i}$ and functional parameters $x_{j}^{\left(F\right)}=\theta_{j}$ that indirectly, or rather functionally, encode the target pattern by parametrizing the intercellular communication and intracellular information processing competencies of the NCA that facilitate the self-orchestrated pattern formation process.

If no ANN at all were present in our model, i.e., $\theta_{j}=\left\{\right\}$, and in the absence of noise $\xi_{c}=0$, we would re-establish a direct mapping between the genotype and phenotype as $c_{i}\left(0\right)=c_{i}\left(t_{D}\right)$, and thus a direct encoding of the target cell type pattern could be achieved $g_{i}\left(0\right)=g_{i}\left(t_{D}\right)$. However, by default, we allow each cell to successively regulate its own cell state towards a target homeostatic value via an iterative perception–action cycle defined by Equation (1) and, moreover, to communicate in that way with neighboring cells. More specifically, each cell updates its cell state solely based on its own and the states of its adjacent neighbors, which, in turn, update their states based on their respective local environment. We explicitly avoid direct environmental feedback to the cells’ perception (such as an individual or collective reward signal) but fully restrict the NCA to intercellular communication (via cell state updates) and intracellular information processing. These uni-cellular agents thus exhibit a certain level of problem-solving competencies that can be utilized for the challenge at hand, in our case, for a collective system-level objective of forming a specific two-dimensional target pattern [95,101,102].

**Figure 3:**
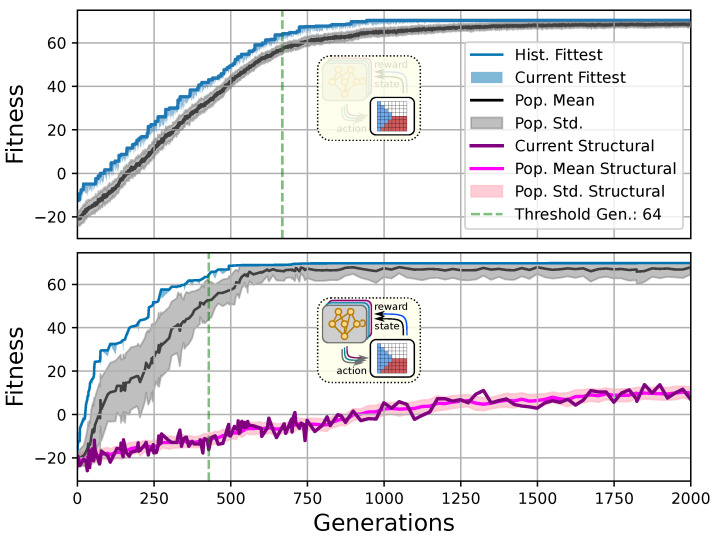
Typical fitness trajectory over several generations of CMA-ES [103] of an NCA-based 8×8 Czech-flag morphogenesis task without (top) and with competency (bottom), corresponding to (i) direct and (ii) a multi-scale competency encoding of the target pattern as discussed in the text, representative of related experiments at similar system parameters (cf. Figure 4). We present the historically- (blue) and currently best fitness value per generation (light blue), and the mean (black) and variance (gray) of the fitness of the entire population. Moreover, the current structural fitness (purple), the mean structural fitness of every generation (magenta), and the corresponding standard deviation (light-pink area) are presented; in the top panel, the structural and phenotypical fitness is equivalent, and thus only the latter is shown. The task is solved when a final fitness score of $F_{j}=64$ is reached (marked by the green dashed line), i.e., when 8×8=64 cell types are correctly assumed after $t_{D}=25$ developmental steps. The cartoon insets represent the perception–action cycle of the NCA, assembling an initial (random) arrangement of cell types into the target pattern; for the direct case (top panel), the NCA ANN is disabled, which is illustrated by masking the agential parts in the cartoon.

With the explicit partitioning of the genome into a structural part, i.e., $x_{j}^{\left(S\right)}$, and a functional part, i.e., $x_{j}^{\left(F\right)}$, we can study the effect of direct vs. multi-scale, or the competency-driven encoding of phenotypic traits in the process of evolution, and, moreover, quantitatively tackle the question of whether *competent parts affect the process of evolution and evolvability*. In any case, the initial cell state pattern is given by the structural part of the genome. Thus, in the absence of noise and without any active functional part in the genome, the set of initial cell states directly represents the final pattern, while otherwise, cell states can either be modified passively by noise in the system or actively through actions by the cells during the developmental stage. Thus, we employ CMA-ES [103] to either evolve the (i) structural, or both (ii) the structural and functional parts of the genome of an NCA simultaneously with the shared objective of self-assembling an 8×8 Czech-flag pattern in $t_{D}=25$ developmental time steps in the presence of noise $\xi_{c}=0.25$ (cf. Section 3.2 and Section 4.1 for details). More explicitly, in case (i), we restrict the cell state update of the NCA by disabling all cell actions $a_{i}\left(t_{k}\right)\to a_{i}^{*}\left(t_{k}\right)=0$ but formally keep the functional part of the genome in the evolutionary search. In turn, we allow the NCA in case (ii) to afford both the structural and functional parts of the genome, thus giving the evolutionary process the opportunity to prioritize one over the other. We thus bias the evolutionary process in case (i) to effectively search the space of direct phenotypic encodings, while keeping the search space dimensions balanced in both cases. The results of this experiment are presented in Figure 3.

We can see in Figure 3 that both the evolution of the (i) direct and (ii) multi-scale encoding schemes of the target pattern can be achieved with the presented framework, and a fitness threshold of $F_{j}=64$ is reached after ≈300–600 generations, thus solving the problem. However, depending on the encoding scheme (i) or (ii), we can identify clear qualitative differences in the strategy and the “efficiency” of the evolutionary process, i.e., how many generations it takes to reach a certain fitness threshold and eventually converge (cf. top and bottom panel of Figure 3, respectively). The respective fitness score of the direct case (i) grows steadily and almost monotonically over successive generations until the threshold of $F_{j}=64$ is reached after 668 generations for that particular run, and the EA converges at a maximum fitness of $\max F_{j}^{\left(i\right)}=70.25$ after 942 generations (see Section 4.1 for details on the threshold fitness values). In contrast, the evolutionary process of the multi-scale case (ii) undergoes significant leaps as reflected by the corresponding fitness score, which can increase rapidly if a suitable innovation, i.e., a favorable crossover or mutation event in the functional parameters $\theta_{j}$, occurs; the initial standard deviation of the fitness of the entire population is significantly larger compared to the direct case (i), yet the threshold fitness of $F_{j}=64$ is reached in 428 generations, and the EA converges after 679 generations (although at a lower maximum fitness of $\max F_{j}^{\left(ii\right)}=69$).

The results presented in Section 4.2 are based on selected evolutionary optimization runs that are representative of related experiments with similar parameterizations. However, one should keep in mind that such results are always susceptible to chance in initial conditions or mutations in the EA but also to developmental noise; moreover, the hyperparameters of the evolutionary search or even the specific ANN architectures can influence the evolvability of such NCA systems. Thus, we present in Section 4.3 below a more statistically significant analysis of the evolutionary implications of direct and multi-scale encodings under various conditions of the cellular agents’ competency levels and the developmental noise.

Our separation of the genotype $x_{j}$ into a structural $x_{j}^{\left(S\right)}={\left\{c_{i}\left(0\right)\right\}}_{j}$ and into a functional part $x_{j}^{\left(F\right)}=\theta_{j}$ moreover allows us to extract the structural (or genotypic) fitness along an entire evolutionary history: we define the structural fitness as the fitness score $r(p_{j}^{*})$ of a phenotype $p_{j}^{*}$ with evolved structural genes ${\left\{c_{i}\left(0\right)\right\}}_{j}$ but with disabled agency $a_{i}\left(t_{k}\right)\to a_{i}^{*}\left(t_{k}\right)=0$. Notably, in the direct case (i), we have $p_{j}=p_{j}^{*}$, which is illustrated in Figure 1A and reflected in the top panel of Figure 3; the structural fitness of the multi-scale case (ii) is explicitly visualized in the bottom panel of Figure 3. In the latter case, the structural fitness remains essentially detached from the phenotypic fitness $p_{j}^{*}\ll p_{j}$ during the entire evolutionary history (which also explains the convergence to a suboptimal maximal fitness level of $\max(F_{j})=69$ in this particular NCA solution, as the final Czech flag pattern first needs to be assembled from the corresponding imperfect initial cell configurations $x_{j}^{\left(S\right)}$). This all suggests that in contrast to (i), the EA in (ii) can make the most use of exploring the functional part of the genome, i.e., the space of behavior-shaping signaling and information processing [1], and, in turn, that the mere presence of competent parts drastically changes the search space accessible to evolution [3]; to show this explicitly, we present in Appendix E an illustration of the evolution of the morphogenesis process.

Interestingly, we still observe a slow but steady increase in the structural fitness in the long term in case (ii), owed to the small additional reward signal $r_{T}$ reinforcing the cellular agents to maintain the target pattern over time. This can most efficiently be achieved if the agent starts from a perfect set of initial cell types, representing a particular sub-space in the parameter space that might not necessarily be easily accessible to the EA at all stages during the evolutionary search. However, we would like to stress that such a slow transfer of problem-specific competencies from an agential, highly adaptive functional part $x_{j}^{\left(F\right)}$ to a rather rigid structural part $x_{j}^{\left(S\right)}$ of the genome could be a manifestation of the Baldwin effect [14].

While remaining neutral with respect to the system level fitness score, this competency transfer seems to affect the entire population presented in Figure 3 as reflected by the successively increasing population-averaged structural fitness score. Notably, and as detailed in Appendix F, we identified an associated decrease in the robustness against increasingly noisy cell state updates of the corresponding solutions with larger structural fitness. This suggests a reduction in uni-cellular competencies and might relate to the “paradox of robustness” discussed in Refs. [51,52,53,121,122,123,124,125,126,127]. Through a computational lens, such a competency transfer would also allow, as soon as the structural part of the genome is reliable enough, to repurpose the system’s competency to adapt to other independent tasks, and thus may facilitate the, in biology, ubiquitous effects of adaptability and polycomputing in related systems [128].

This all illustrates that an agential material [1,94], or more precisely, a substrate composed of competent parts, can have significant effects on the process of evolution and evolvability, especially for morphogenesis tasks. We thus conclude that, if competent parts are available, evolution prefers exploiting competency over direct encoding—if the environment requires competency at all (see discussion in Section 4.3). This leads to the conclusion that “competency at the lowest level greatly affects evolution and evolvability at the system level”.

### 4.3. Evolution Exploits Competency over Direct Encoding, if Necessary

Here, we investigate the effects of varying different levels of competency at the cellular level of a multi-scale competency architecture on the evolutionary process of morphogenesis. More specifically, we introduce the *decision-making probability* (I) $P_{D}$, which constrains the ability of each cell individually to perform cell state updates in the environment: $P_{D}$ defines the probability at which a proposed cell state update of each individual cell in the NCA is executed (or otherwise omitted). Thus, varying the decision-making probability from $P_{D}=0$ to $P_{D}=1$ smoothly transitions the system’s behavior from a direct encoding scheme without competency to an increasingly reliable multi-scale competency architecture (cf. Figure 3).

Another, somewhat hidden, level of competency we already discussed in Section 3.1 is each cell’s ANN architecture: An RGRN agent with internal memory can acquire and execute tasks differently than a simpler FF agent without any feedback connections except for its cell state $c_{i}\left(t_{k}\right)$. Comparing the evolutionary implications of such functionally different ANN architectures is, however, not trivial, and is thus kept to a minimum here.

However, we parameterize both FF and RGRN agents such that their controller part of the ANNs (cf. Figure 1C, Section 3.1, and Appendix A) are (II) stacks of *R* redundant copies of the same controller ANN, each copy with its own set of parameters, which take the same pre-processed aggregated sensor embedding as input, and whose individual outputs are averaged into a single action-output of a cell. Inspired by redundancy in error-correcting codes [105,106], we thus allow cells with higher values of this *redundancy number R*, i.e., with many alternative routes through the controller part of the ANN, to—in principle—integrate environmental signals more generally compared to R=1, thus affecting the cells competency.

While scaling from $P_{D}=0$ to $P_{D}=1$ smoothly increases a cell’s competency to reliably regulate its cell state, increasing *R* enhances the computational capacities of the uni-cellular agents. Henceforward, we interpret (I) $P_{D}$ and (II) *R* as two competency levels in our system, which we can vary (I) continuously and (II) discretely.

Analogous to Section 4.1 and Section 4.2, we thus utilize CMA-ES to evolve the genotypic parameters of an NCA to self-assemble the 8×8 Czech flag pattern under different conditions (I–II), and expose the cells to different noise-levels (III) $\xi_{c}$ during cell state updates defined in Equation (1).

**Figure 4:**
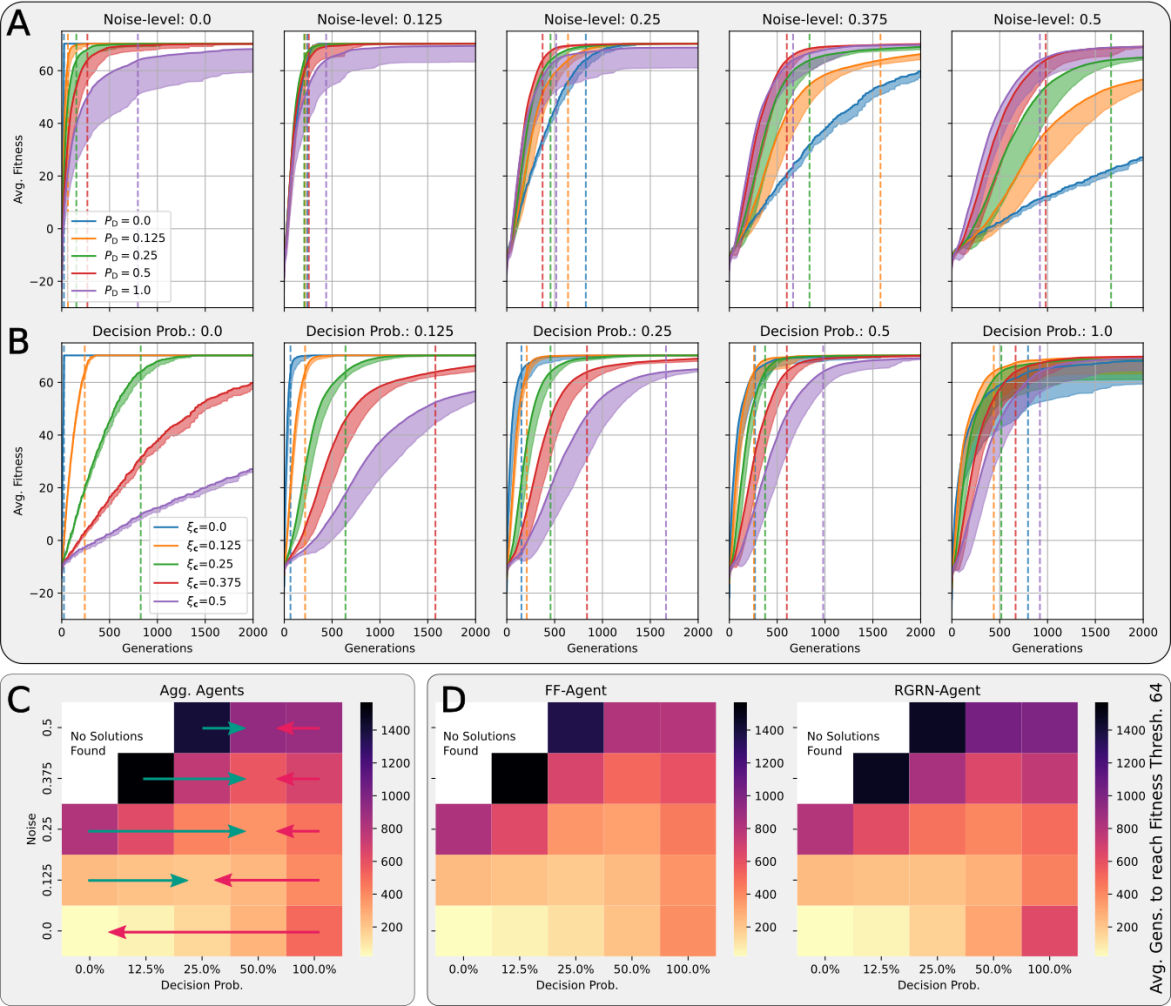
(**A**,**B**) The average fitness per generation of the best-performing individual in a population of 65 independent evolutionary processes of the 8 × 8 Czech flag task, evaluated from left to right at different noise levels (decision-making probabilities) and color-coded by the decision-making probabilities (noise-levels), for panels (**A**,**B**) respectively; solid lines mark average fitness values, the shaded area marks the standard deviation (to lower values only), and dashed lines indicate when an average fitness threshold of 64 is crossed, solving the problem. (**C**) Heatmap of the average generation number when the fitness threshold of 64 is crossed at particular combinations of the decision-making probability and noise level as detailed in (**A**,**B**); green and red arrows respectively indicate directions along $P_{D}$ of increasing and decreasing values of the average fitness at fixed noise values. (**D**) Same as (**C**) but partitioned by the respective FF-agent or RGRN-agent architectures used in the respective CMA-ES runs.

In Figure 4A,B, we present the corresponding fitness scores of a maximum of 2000 generations of CMA-ES for different noise levels $\xi_{c}\in \left[0,0.5\right]$, averaged over different values of the decision-making probability $P_{D}\in \left\{0,12.5\%,25\%,50\%,100\%\right\}$ for both FFagents and RGRN agents. Moreover, for each realization of $\xi_{c}$ and $P_{D}$, we utilize experiments with different redundancy numbers R∈{1,2,4,8,16} and employ 15 statistically independent EA runs for each parameter combination $\xi_{c}$, $P_{D}$, and *R*, and thus arrive at 75 statistically (and functionally, with respect to an agent’s ANN architecture) independent fitness trajectories per ($P_{D}$, $\xi_{c}$) combination; see Section 4.1 and Appendix D for more details on the EA parameters. In Figure 4C, we present the average number of generations it takes to solve the problem (to reach a fitness threshold of $F_{j}=64$) for each combination of $P_{D}$ and $\xi_{c}$, aggregated over the agents’ ANN architectures, FF or RGRN, and the respective redundancy numbers *R* for 15 statistically independent EA runs each; in Figure 4D, we present the data from Figure 4C but separately for both ANN architectures.

We observe in Figure 4 that, depending on these two parameters $P_{D}$ and $\xi_{c}$, for no- or very low noise levels $\xi_{c}\approx 0$, the evolutionary search is most efficient, i.e., finds the solution in the fewest number of generations, on average, for low values of the competency level $P_{D}\approx 0$. Thus, in these situations, direct encoding (achieved via $P_{D}=0$) seems to be preferable to competency-driven encodings with $P_{D}>0$ (as indicated by the bottom red arrow in Figure 4C); this is partly owed to the specific definition of the cell types $g_{i}\left(t_{k}\right)$ given by Equation (2), making a noiseless search very simple for the EA. However, for more realistic, noise conditions $\xi_{c}>0$, the situation changes drastically. With increasing the noise level, the evolutionary efficiency of NCAs with higher competency levels is significantly greater compared to those with low competency levels, especially for the direct encoding scheme (as indicated by the green arrows in Figure 4C); for noise levels of $\xi_{c}=0.375$ and 0.5, the EA does not even find solutions for the direct encoding case with $P_{D}=0$ in 2000 generations, as cell state updates become increasingly necessary to counteract the noise in the system. There is a clear trend of increasing the evolutionary efficiency in our in silico morphogenesis experiments by increasing the competency level for increasingly difficult environments with high noise levels.

Thus, we conclude that scaling competency has a strong effect on the process of evolution, and in realistic situations (with moderate to high noise), competency may greatly improve the evolutionary efficiency and evolvability of collective self-regulative systems.

**Figure A8:**
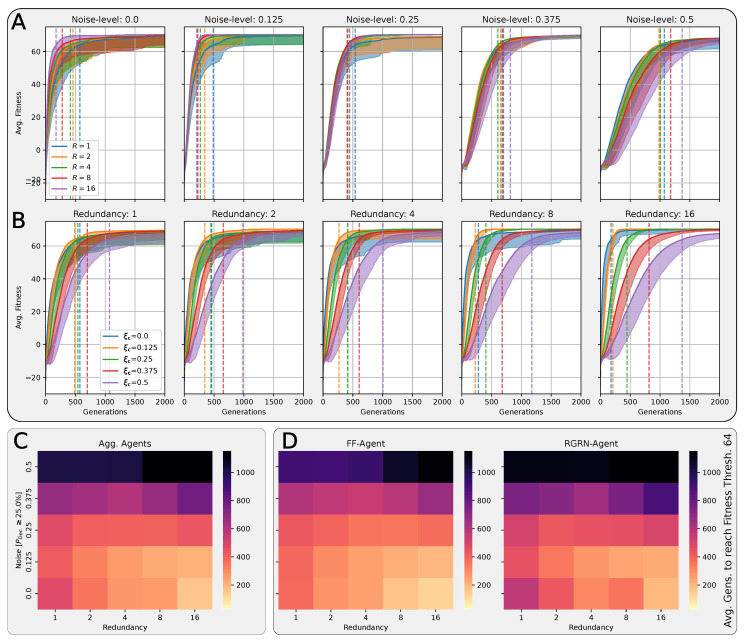
Similar to Figure 4 but presented for the redundancy number *R* vs. noise level $\xi_{c}$ and aggregated over all values of the decision-making probability $P_{D}\geq 0.25$. (**A**,**B**) The average fitness per generation of the best-performing individual in a population of 65 independent evolutionary processes of the 8 × 8 Czech flag task, evaluated from left to right at different noise levels (redundancy numbers) and color-coded by the redundancy numbers (noise-levels), for panels (**A**,**B**) respectively; solid lines mark average fitness values, the shaded area marks the standard deviation (to lower values only), and dashed lines indicate when an average fitness threshold of 64 is crossed, solving the problem. (**C**) Heatmap of the average generation number when the fitness threshold of 64 is crossed at particular combinations of the redundancy number and noise level as detailed in (**A**,**B**). (**D**) Same as (**C**) but partitioned by the respective FF-agent or RGRN-agent architectures used in the respective CMA-ES runs.

It might be noteworthy that for evolving the 8×8 Czech flag pattern, essentially no qualitative difference in the evolutionary efficiency between FF agents and RGRN agents with the given number of parameters was observed. Also, the evolutionary implications of utilizing a number of R>1 redundant copies within the controller ANNs of the cells of an NCA is much less pronounced, compared to the results depicted in Figure 4 as can be seen in Figure A8 of Appendix H. However, for more advanced problems such as assembling a 9×9 smiley-face pattern (see Appendix G), RGRN agents seem to outperform simpler FF agents significantly in terms of evolutionary efficiency. Moreover, a larger redundancy number of R≥4 is required by the evolutionary process to more efficiently evolve the functional parameters of an NCA compared to a direct encoding scheme, hinting at a capacity bottleneck of the deployed ANNs.

### 4.4. There Is a Trade-Off between Competency and Direct Encoding Depending on Developmental Noise

**Figure 5:**
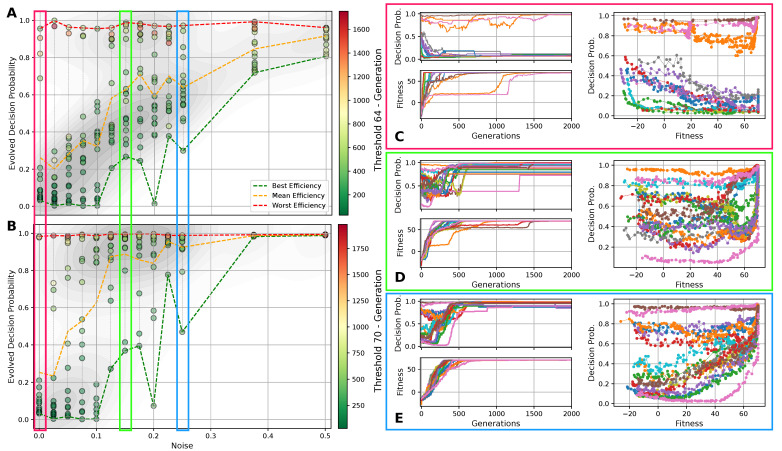
(**A**) The evolved decision-making probability $P_{D}$ for different noise levels $\xi_{c}$ when a fitness threshold of 64 for the 8×8 Czech flag task is reached; each symbol represents an independent lineage with a color-coding that indicates the number of generations it took for that particular lineage to cross the specified fitness threshold. The green/orange/red dashed lines indicate at which value of $P_{D}$ the evolutionary process crossed the fitness threshold the fastest/on average/the slowest (i.e., in the least, average, or largest number of generations) for each noise level. (**B**) Same as (**A**) but with a fitness threshold of 70. For both (**A**,**B**), the red/green/blue frames emphasize the noise level $\xi_{c}=0,0.125$ and 0.25 corresponding to panels (**C**–**E**), respectively: The latter show the evolution of the decision-making probability/fitness (top/bottom left panel) and the value of the decision-making probability as a function of the corresponding fitness during the evolutionary process of each lineage (right panel) for all lineages (indicated via color-coding) at the specified noise level. Results are shown for an RGRN-agent architecture with redundancy R=1, and are qualitatively similar to those of an FF-agent architecture.

A careful analysis of the results shown in Figure 4 reveals that the largest competency level of $P_{D}=1$ does not result in the highest evolutionary efficiency for any presented noise level. On the contrary, populations with slightly lower competency levels of $P_{D}=0.5$ or even $P_{D}=0.25$ perform best at noise levels $\xi_{c}\in \left\{0.25,0.375,0.5\right\}$ and 0.125, respectively (as indicated by the green and red arrow ends in Figure 4C). In fact, cells with an initially random genome (comprising the ANN and initial cell state parameters) that are forced to make “uninformed”, i.e., initially random, decisions at every time step can interfere with the performance of the EA, as even initially perfect cell state configurations will be destroyed during such a randomized developmental stage. We suspect that this leads to corresponding delays in the evolutionary search compared to situations where populations can better rely on the structural part of the genome. Indeed, populations with “overconfident” actions can be trapped in local optima for many generations at all stages of the EA, which, in our system, may only be resolved by very specific but random mutations of the functional part of the genome (as we show later through Figure 5 in Section 4.4). This is reflected in Figure 4A,B by the large deviations in the average fitness trajectories for large $P_{D}$ values.

The insights from the above lead to the questions of whether there is a “natural” or optimal competency level, with respect to the decision-making probability $P_{D}$, or whether a mutable competency level can be utilized by the evolutionary process to improve the efficiency of guiding a population towards high-fitness regions in the parameter space. Thus, we include the decision-making probability as an additional *competency gene* $x_{j}^{\left(C\right)}$ into the NCA genome $x_{j}\to x_{j}=x_{j}^{\left(S\right)}\cup x_{j}^{\left(F\right)}\cup x_{j}^{\left(C\right)}$, cf. Equation (3), and we perform in silico morphogenesis evolution experiments of the 8×8 Czech flag pattern for different noise levels $\xi_{c}$, analogous to Section 4.3. We analogously limit the numerical range of the competency gene $x_{j}^{\left(C\right)}$ to the interval [−3,3], and extract the corresponding decision-making probability via $P_{D,j}=\frac{1}{2}\left(\tanh\left(x_{j}^{\left(C\right)}\right)+1\right)$. Notably, for the experiments shown in this sub-section, we use $L_{2}$ regularization on the genotypic parameters $x_{j}=\left(x_{j,1},\cdots ,x_{j,N_{x}}\right)$ through subtracting $r_{L_{2}}\times \sum_{i=1}^{N_{x}}x_{j,i}^{2}$ from the fitness score defined in Equation (5), with $r_{L_{2}}=0.01$ (the $L_{2}$ regularization applied to $x_{j}$ does not introduce a bias between the minimal $P_{D,j}=0$ and maximal $P_{D,j}=1$ competency levels, as the $L_{2}$ regularization is applied to $x_{j}^{\left(C\right)}$, not $P_{D,j}$; both $P_{D,j}$ and the $L_{2}$ regularization are symmetric with respect to the sign of $x_{j}^{\left(C\right)}$).

In Figure 5A,B, we present the evolved competency level for different noise levels after fitness thresholds of 64 and 70 are crossed, respectively, for 10 independent lineages per noise level for an RGRN architecture. The problem is considered solved at a fitness of 64, but since we reward the NCAs to maintain the target pattern over time via $r_{T}$ in Equation (5), a higher maximal fitness score of 70.25 can be reached after $t_{D}$ developmental steps for a sufficiently long evolution. Thus, we here relate Figure 5A to the evolutionary stage of having achieved the process of morphogenesis, and Figure 5B of having achieved morphostasis. For both cases, we essentially see two strategies emerging (see also Figure 5C–E): (i) one, where competency is maximized very early during the evolutionary process that then remains near the maximally possible value of $P_{D}=1$, and (ii) a hybrid strategy where a significantly lower competency level is assumed that still allows to solve the problem.

Notably, strategy (i) is predominantly pursued at high noise levels, where large cell state fluctuations in the environment favor informed actions by the cellular agents. In contrast, the second strategy (ii) emerges more frequently in lineages evolved at low noise levels where, especially at very low noise levels $\xi_{c}\approx 0$, most of the evolutionary processes result in solutions that avoid competency altogether, and a direct encoding scheme ($P_{D}$ = 0) is evolved. Intermediate competency levels evolve in the corresponding intermediate noise regime. Following the trend of evolving morphogenesis (by crossing a fitness score of 64) to morphostatsis (by converging to the maximal fitness value of ≈70) in Figure 5A through B, we see that the two strategies, (i) and (ii), “sharpen” during the course of the evolutionary process such that $P_{D}$ predominantly converges to the minimally or maximally possible values of 0 and 1, depending on the environmental conditions.

We also illustrate the evolved competency level of the particular lineage at all noise levels in Figure 5A,B, at which the respective fitness threshold is crossed in the minimal and maximal number of generations (and on average) amongst all 10 independent lineages per noise level. This clearly reveals that evolutionary processes that follow a more direct encoding strategy (ii) can evolve the problem at hand efficiently—if this is permitted by the developmental noise. However, when increasing the noise level, the evolutionary process can afford to evolve—or put differently, increasingly relies on evolving—the multi-cellular intelligence of the NCA to perform morphogenesis and morphostasis, thus following a third strategy (iii) that integrates both strategies (i) and (ii) in a non-trivial way. We observe in Figure 5A that the most efficient strategy for evolving morphogenesis seems indeed to be such a hybrid approach (iii), where a minimally necessary competency level is utilized at a specific noise level such that the corresponding evolutionary process can, again, be very efficient in solving the task.

Moreover, this also holds for the stage where morphostasis is reached, cf. Figure 5B: lineages that efficiently evolved to solve morphogenesis in our experiments also (typically) evolve to solve morphostasis efficiently. To emphasize this, we present in Figure 5C–E the “temporal dynamics” of the population-wise highest fitness and the corresponding competency level per generation for all lineages at selected noise levels $\xi_{c}=\left\{0,0.125,0.25\right\}$; we also present for all corresponding lineages that have been evolved at these selected noise levels the genotypic competency level $P_{D,j}$ against the corresponding phenotypic fitness scores $r_{j}$, and we find an apparent yet non-trivial relation between these two quantities: typically, an initial rise in fitness $r_{j}$ in early generations is associated with a decline in $P_{D,j}$ which is more pronounced at lower noise levels. For intermediate noise levels $0<\xi_{c}\ll 1$, we find that $P_{D,j}$ often assumes a minimum (i.e., a minimally required yet finite competency level) when the evolutionary process reaches a fitness level of ≈64. We suspect that this allows the evolving morphogenetic process to establish good starting configurations based on changes in the structural genome, which can most efficiently be performed at a minimal(ly necessary) competency level given a certain developmental noise level in the environment. However, the competency is then quickly pulled towards a maximum level of $P_{D,j}=1$ when the EA converges at a maximum fitness score of ≈70, at the morphostasis stage. For large noise levels, e.g., $\xi_{c}=0.25$ as depicted in Figure 5E, the competency level rises with the corresponding fitness score in a much more monotonic way, emphasizing the necessity of the corresponding NCAs to utilize the cellular competency to solve the problem already at an early stage of the evolutionary process.

Curiously, we also see lineages that settle at the highest possible competency levels throughout their evolutionary history, even in conditions without noise as can be seen in Figure 5C: here, an initial “frozen accident” may cause an entire lineage to maintain high competency levels due to a lack of diversity in the corresponding gene, although this is not even necessary to solve the task. However, these high competency levels early on during the evolutionary process can cause the population to stagnate at sub-optimal regions in the parameter space for many generations if the corresponding policy of the cells is sub-optimal but rigid to strategy changes via small mutations in the genome. The population seems “trapped” until a favorable mutation or crossover event occurs in the functional part of the genome of an individual that guides the entire population towards higher fitness scores, eventually solving the problem. We suspect that this is also the reason for the lower evolutionary efficiency of the “most competent” configurations (with $P_{D}=1$) compared to the slightly less competent cases (with $P_{D}=0.5$) of the experiments depicted in Figure 4 [129].

Thus, we conclude, that if the evolutionary process can afford to evolve its own competency level, there seems to be a trade-off—during the entire course of the evolutionary process—between “going direct” or “going competent”, depending on the developmental noise. Moreover, the randomly initialized starting conditions may favor either direct or multi-scale encoding strategies, which may not only affect the “final” competency level that the evolutionary process converges to but can also greatly influence the efficiency of the evolutionary process itself. In general, the most efficient strategy for evolving morphogenesis tasks seems to be a non-trivial trade-off between finding a suitable initial cell state configuration that then allows the competency-based self-assembly of the target pattern to “kick in” and solve the task efficiently.

### 4.5. Competency Can Lead to Generalization

**Figure 6:**
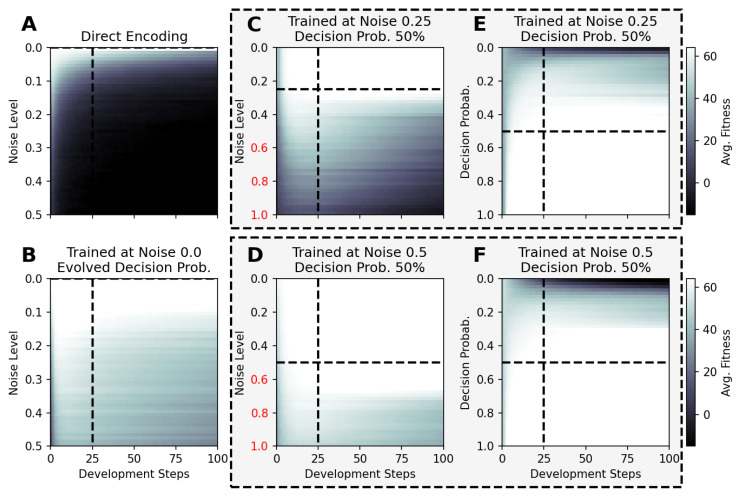
The average fitness score of 100 independent evaluations of selected NCA results utilized at noise (**A**–**D**) and competency-level conditions (**E**,**F**), which have not been experienced during training for an increased total lifetime of 100 time steps. The respective NCAs have been evolved at zero-noise without competency (**A**), with evolvable competency (**B**), and under different noise conditions and decision-making probabilities (**C**–**F**), with a fixed number of $t_{D}=25$ developmental steps; the results of all panels except for (**B**) are based on RGRN-agent architectures with the training conditions given by titles and dashed lines. The data presented in panels (**C**,**E**) and (**D**,**F**) are respectively based on the same NCA solution (indicated by the dashed frames), while the noise level is varied in (**C**,**D**) at a fixed competency level of $P_{D}=0.5$, and the competency level is varied in (**E**,**F**) at a fixed noise level of ($\xi_{c}=0.25$, $\xi_{c}=0.5$)], respectively.

We are ultimately interested in the question of whether a substrate of competent parts shows the ability to generalize to environmental conditions that have never been experienced by its evolutionary predecessors, and hence would allow the evolutionary process to adapt an organism to changing environmental conditions more efficiently compared to a direct encoding scheme. Thus, we systematically vary in Figure 6 the system parameters, i.e., the noise level and the decision-making probability competency level, for selected NCA solutions of the Czech flag problem that have been trained with certain sets of the system parameters above.

For instance, we utilize NCA solutions that have been evolved to solve the 8×8 Czech flag problem in $t_{D}=25$ developmental steps (see above) under zero-noise conditions without and with evolvable competency. Here, we utilize such solutions for larger noise levels of $\xi_{c}\in \left[0,0.5\right]$ and for lifetimes of 100 time steps and present the average fitness values of 100 statistically independent simulations at each particular noise level in Figure 6A,B, respectively, without any further evolutionary optimization. Analogously, we expose NCA solutions that have evolved with a competency level of $P_{D}=0.5$ and noise levels of $\xi_{c}=0.25$ and 0.5, respectively, to vastly different noise levels of $\xi_{c}\in \left[0,1\right]$ compared to the conditions during their respective evolutionary processes, and present the results in Figure 6C,D. Eventually, we again deploy the latter NCA solutions but vary the competency level $P_{C}\in \left[0,1\right]$ instead, at respectively fixed noise levels of $\xi_{c}=0.25$ and 0.5, with the results depicted in Figure 6E,F. Notably, we only consider the “correctness” part of the fitness score, i.e., the first term in Equation (5) by setting $r_{T}=0$ and $r_{S}=0$.

The results in Figure 6 demonstrate that the performance of the here evolved NCAs, optimized with evolutionary methods to assemble and maintain a target morphology over time at particular system parameters, differs greatly between NCA solutions that follow the direct- or multi-scale encoding paradigms when subjected to novel environmental conditions: The typical fitness over the lifetime of an NCA without competency that encodes the target phenotype pattern directly (cf. Figure 6A) is constantly affected by random fluctuations and thus decreases in fewer time steps with increasing noise levels in a diffusive process; the duration of how long the corresponding maximum fitness score of 64 can be maintained and the speed at which the fitness eventually decays during the lifetime of the here discussed 8×8 Czech flag NCA depend on the particular noise level and on the values of the initial cell states, which are limited numerically to the interval [−3,3] for each cell. In contrast, NCA solutions with larger competency levels that have been evolved at finite noise-level conditions still perform well—and can maintain the target pattern for exceptionally long times—also when changing the system parameters dramatically (cf. Figure 6B–F); note the noise-level axis of $\xi_{c}=0$ to 1, compared to the maximum noise levels of $\xi_{c}=0.5$ during training.

The results in panel Figure 6B are especially curious, as the corresponding NCA has been trained to evolve its decision-making probability alongside the structural and functional parts of the genome at zero noise conditions. While no competency at all would have been required to solve this task, the presented NCA solution evolved to afford a maximum competency of $P_{D}=1$ (cf. Figure 5C). Strikingly, this particular NCA is capable of resisting much larger noise levels of $\xi_{c}\approx 0.25$ while maintaining the pattern perfectly for at least $t_{D}=25$ steps, and the average fitness score of 100 independent solutions does still not drop below a certain threshold of ≈40–50 for even higher noise levels and for 100 time steps. Notably, there appears to be a bifurcation of the long-term behavior of these NCA solutions (not shown here) where the NCA—in some realizations—maintains the target pattern perfectly for long times, while in other independent runs, the fitness drops quickly.

In this sub-section, we thus show that NCAs that have evolved to assembly and maintain a target pattern within a relatively short developmental stage are capable of maintaining the corresponding target pattern over much longer time scales—without any further optimization—and thus show great signs of functional, morphostatic generalizability. Moreover, the here-discussed in silico morphogenesis and morphostasis model systems are capable of handling, essentially on the fly, system–parameter combinations that neither they nor their evolutionary ancestors ever experienced before. Thus, we conclude that such multi-scale competency architectures [1], whose substrate is composed of competent rather than passive parts, can be more than capable of generalizing to changes in their environment—within reasonable boundaries, of course—by allocating robust problem-solving competencies at many scales [93,94].

### 4.6. Competency Can Augment Transferability to New Problems

Deducing from the discussion in Section 4.5 about the generalizability of multi-scale competency architectures [1] towards changing environmental conditions, such systems should also exhibit increased evolvability and transferability properties to new problems: if such multi-scale competency architectures are capable of adapting their behavior towards changing environmental conditions on the fly during a single lifetime (cf. Figure 6), this has great consequences for the evolutionary process when environmental conditions change.

Thus, we utilized the NCA solution discussed in Figure 5C and Figure 6D and performed subsequent CMA-ES on the 8×8 Czech flag problem at changed environmental conditions, i.e., at higher noise levels: only a single or, at most, a handful of generations are necessary for solving the task even at intermediate and high noise levels of $\xi_{c}=0.25$ and 0.5.

To emphasize the potential of the transferability of multi-scale competency architectures, we here investigate the adaptation capability of pre-evolved NCAs when their objective function is suddenly changed, i.e., when the environment starts selecting for different target patterns than the one they have originally been evolved for. More specifically, we utilize NCA solutions from Section 4.3, and discussed in Figure 4, which successfully solve the 8×8 Czech-flag task, and additionally perform 1000 evolutionary cycles of CMA-ES on a related 8×8 blue-, white-, red-, Viennese-, blue/white-, and blue/red-flag morphogenesis task for various noise and competency levels. We allow changes to both the structural and functional parts of the genomes of the pre-evolved NCA.

**Figure 7:**
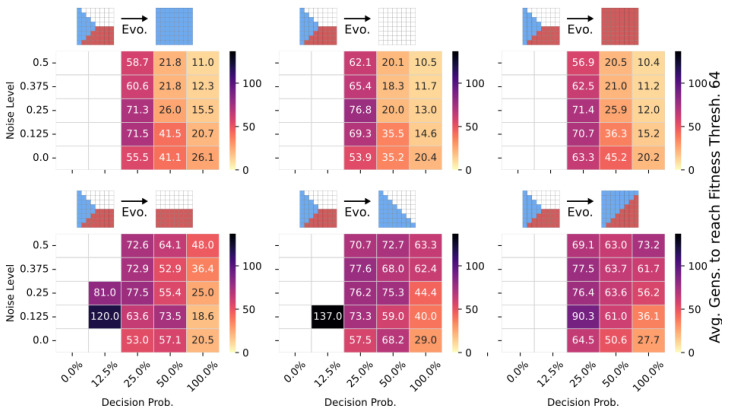
The average number of generations it takes for the CMA-ES to adapt a pre-evolved NCA solution that can solve the 8×8 Czech-flag morphogenesis task to adapt, respectively, to the 8×8 blue-, white-, red-, Viennese-, blue/white-, and blue/red-flag morphogenesis tasks instead (cf. panel insets) and reach a correctness fitness score of 64. We specifically adapt Czech-flag NCA solutions that have been pre-evolved at a noise level of $\xi_{c}=0.25$ but with corresponding competency levels according to the horizontal axis in Figure 4, and deploy CMA-ES for 1000 generations at the corresponding noise/competency levels depicted here on the vertical/horizontal axis, and average over multiple CMA-ES runs and corresponding redundancy numbers R=1,2,4,8,16.

In Figure 7, we present the corresponding number of generations it takes for 10–60 CMA-ES runs on average to adapt a pre-evolved, i.e., “informed”, NCA solution that can solve the 8×8 Czech-flag morphogenesis task to then solve the respective new morphogenesis task under different environmental conditions. We see a clear advantage in terms of the evolvability and adaptability of pre-evolved individuals at high-competency levels (in contrast to individuals with lower competency levels) so that adaptation can happen in as few as ≈10 generations. While the Czech→blue-, white-, and red-flag tasks are rather trivial (see top panels in Figure 7), computationally, the Czech→Viennese-, blue/white-, and blue/red-flag adaptation tasks (bottom panels in Figure 7) are more complicated. Still, the latter can be solved in as few as ≈20 generations compared to ≫100 generations of evolving a corresponding randomly initialized NCA to solve the Czech-flag problem from scratch as shown in Section 4.2 and Section 4.3.

Thus, we conclude that pre-evolved (or “informed”) competency at subordinate scales of a multi-scale competency architecture greatly enhances a collective system’s capability of adaptation. Thus, a competent and informed substrate has great effects on a multi-scale competency architecture’s evolvability towards changing environmental conditions and on the transferability of already acquired (evolved) solutions to new problems.

## 5. Conclusions

We have investigated the evolutionary implications of multi-scale intelligence on an example of the in silico morphogenesis of two-dimensional tissue of locally interacting cells that are equipped with tunable decision-making machinery. More specifically, we have utilized evolutionary algorithms (EAs) [103] to evolve the parameters of neural cellular automata (NCAs) [100] on morphogenesis tasks under various conditions of the competency level of the uni-cellular agents and the developmental noise in the system.

In this model of a multi-scale competency architecture [1], a two-dimensional grid of locally interacting cells is tasked to self-assemble and maintain a global spatial target pattern of predefined cell types, here, primarily of a two-dimensional, 8×8 Czech flag pattern (we model and investigate the evolution of the process of morphogenesis and morphostasis in silico and deploy our framework to a self-orchestrated pattern formation task, primarily of a two-dimensional 8×8 Czech flag pattern but also for other much more involved target shapes, such as a 9×9 smiley face (see Appendix G)). Each uni-cellular agent’s internal decision-making machinery is modeled by an artificial neural network (ANN), allowing these cells to independently perceive the cell states of their adjacent neighbors on the grid and propose actions to regulate their own cell state over time via local communication rules. Both the ANN parameters and the initial cell states of all permanent cells represent the parameters of the NCA and are optimized by EAs for a specific in silico morphogenesis task, thus forming the functional and structural part of the system’s genome, respectively.

To investigate the effects of competency in a multi-scale competency architecture on the underlying evolutionary process, we introduce (I) a “competency level” parameter that controls the reliability of the uni-cellular agents of an NCA to regulate their cell types during a noisy developmental stage. This allows us to continuously scale the NCA competency level from a direct encoding scheme of the target pattern (no competency) to a multi-scale competency architecture that self-assembles the pattern with perfect reliability in cell decision executions. Furthermore, we introduce (II) a variable number of redundant sub-modules in the NCA ANN, which we utilize as another “axis” of redundancy- and computational capacity-based competency of the cells’ decision-making machinery.

In large-scale simulations, we systematically vary these two competency levels (I, II), expose the corresponding NCA to different noise conditions (III), and perform several statistically independent evolutionary searches at each parameter combination (I–III). In that way, we demonstrate that an evolutionary process proceeds significantly more rapidly (on average) on noisy pattern formation tasks when evolving the parameters of a multi-scale competency architecture compared to evolving the target pattern directly (with no competency involved).

Our multi-scale competency architecture model and the corresponding evolutionary optimization process comprise several scales: At the smallest scale (1), each structural and functional gene is represented by a floating point number. The functional genes parameterize the behavior of artificial neurons (2), our atomic decision-making centers, which are then hierarchically arranged into layers of artificial neurons (3), sub-modules of interconnected layers (4), to an ANN with a predefined architecture (5). Thus, even the uni-cellular phenotypes (6) in our system—ANN-based agents that maintain a particular internal cell state—are composites of smaller (proto-competent) decision-making centers down the hierarchical ladder. The composite uni-cellular agents perceive the cell states of their grid neighbors (7) on the NCA, perform potentially several cycles of internal calculations, and eventually update their own cell state in a single developmental step. In that way, clusters of different tissue types (8) may be formed in successive developmental steps. A fixed number of developmental steps comprise the lifetime of a single NCA, giving rise to self-assembled phenotypic tissue of cell types on the entire grid of the NCA (9), e.g., as in our case, to the Czech flag pattern. The quality of each individual in an evolutionary population of NCAs (10) is evaluated via a phenotypic fitness score, quantifying the deviation of the assumed cell types from a target pattern. Based on the fitness scores of a particular generation of NCAs, the genotypes of potentially better-adapted successor generations are successively sampled by the EA, closing the loop (1) and forming the largest scale in our system, an evolutionary lineage (11). Eventually, on a meta-scale (12), we compare the efficiency of the evolutionary process at different system parameters (I–III), i.e., at different competency- and noise levels, by analyzing the fitness trajectories of statistically independent lineages evaluated at the same system parameters.

We demonstrate that especially in the presence of developmental noise, affecting cell state updates during morphogenesis, the evolutionary process favors a multi-scale competency-based realization over a direct encoding scheme of the target pattern. Moreover, when the competency level itself is left as an evolvable parameter to the EA, there appears to be a non-trivial dynamical trade-off in the evolutionary process’ efficiency between exploiting the competency level of its components or the direct, pre-patterning-like encoding of the target pattern. We thus report that under realistic conditions (i.e., at moderate noise levels), an evolutionary process can be significantly more efficient when working with an agential rather than a passive material [1,94].

Notably, we explicitly omit a reward or fitness feedback from the environment to the NCAs’ uni-cellular agents’ perception, restricting the cells’ decision-making solely to the local communication of cell state updates between grid neighbors. Thus, the cells need to figure out their own communication protocol such that their single-agent decisions align with the global (multi-agent) system-level objectives of assembling the correct target pattern. These uni-cellular competencies are acquired over evolutionary time scales and can be understood as emergent behavior-shaping signaling [1].

On a more technical note, we specifically employ permutation-invariant ANNs as trainable update functions of the NCAs and successfully evolve the corresponding models to perform the here studied pattern formation tasks. We thus show that, contrary to previous assumptions [101,130], a perfect spatial resolution of neighboring cell states in an NCA is not necessary but that a mean-aggregated neighboring cell state can be sufficient for single cells to reliably contribute to the objective of a larger scale collective. Strikingly, we show that such uni-cellular agents do not even need to distinguish between their own states and the states of their neighbors to achieve this task, thus fully integrating into the tissue locally and essentially losing their individuality [93,95].

Also, in contrast to Ref. [101] and similar work, we do not start our morphogenesis experiments from a single “alive” cell but instead evolve the initial cell states of all permanent cells on the grid of an NCA, while the uni-cellular agents are constantly challenged to correct their state from developmental noise (notably, a process reminiscent of the denoising steps of Diffusion Models [131,132,133,134,135]). This allows us to explicitly distinguish between the evolutionary implications of (i) direct and (ii) multi-scale competency-based encodings of the target pattern, where we either constrain the evolutionary process to (i) only evolve the structural part of the genome, or to (ii) evolve both the structural and functional parts simultaneously. Admittedly, the choice of the structural part of the genome limits the scalability of the approach, as the size of the structural genome will grow correspondingly with the number of cells in the system. However, as occurs with biomechanical [136], biochemical [137,138] and bioelectric pre-patterning [8,94,139], the initial states of an NCA of moderate size could be seen as a coarse-grained scaffold, based on which an NCA of a potentially much higher resolution can run its multi-scale competency-based developmental program to self-assemble a high-resolution target pattern [140]. Alternatively, we suggest utilizing a Compositional Pattern Producing Network (CPPN) [120,141] to indirectly encode the initial states of all cells on the grid of an NCA, allowing such a hybrid approach to perform in silico morphogenesis at scale. Unfortunately, it has been proven difficult, if not unfeasible, to exactly reproduce predefined target patterns reliably with the neuroevolution of CPPNs alone [142], which is why we here refrained from this approach; we emphasize, however, that gradient-based methods such as Neural Radiance Fields (NeRFs) [143] to train CPPN-like architectures might be an interesting workaround.

We find that fully evolved NCA solutions, capable of performing the morphogenesis tasks discussed above, show great signs of generalizability toward changing the system parameters, and can—without any further evolutionary optimization or training—handle noise and competency levels that are vastly different from the training conditions. Consequently, this leads to the increased evolvability of such competency-based models to changing environmental conditions: a subsequent evolutionary process can adapt a pre-evolved solution to altered environmental conditions within a handful or sometimes even a single generation. Moreover, we demonstrate that such pre-evolved NCA solutions can even quickly adapt to new, yet related problems. Specifically, we modify the objective function of our evolutionary process from the 8×8 Czech flag task to self-assemble a blue-, red-, white-, Viennese-, diagonal blue/white and blue/red flag instead, respectively. In most of these situations, an adaptation of an existing NCA solution to the new problem can be performed in significantly fewer generations than evolving the initial 8×8 Czech flag task from a randomly initialized configuration. Typically, these adaptations happen faster the larger the competency level of the NCA is, while for the direct encoding scheme (or in situations with low competency), the structural part of the genome is too dominant to allow quick adaptations by the EA. This suggests that multi-scale competency architectures allow the underlying evolutionary process to not over-train on priors, thus augmenting adaptability through a competent substrate.

We conclude that not only can evolutionary processes efficiently utilize and bring forth the intriguing multi-scale problem-solving machines of biological life but that the efficiency of such evolutionary processes, as well as the generalization abilities, evolvability, and transferability of the corresponding phenotypic outcomes, are strongly affected by the level of competency of the underlying agential material. An intriguing open question is whether this implies a positive feedback loop that enhances that quality over time. Judging from the considerable effects of scaling the competency in the here-studied still shallow multi-scale system on a rather simple in silico evolutionary process (i.e., CMA-ES [103]), it becomes increasingly evident that the vastly more complex multi-scale competency architecture of biological life cycles back and thus affects the process of evolution itself.

One of the key opportunities for future work is to apply the ideas explored here in silico to the understanding of biological mechanisms in natural systems, and to the design of new synthetic constructs via bioengineering [144,145]. It is now known that living tissues implement a kind of multi-scale competency architecture [94]. Problem-solving capacities at one scale, for example, the ability to navigate anatomical morphospace despite perturbations (embryogenesis and regeneration), rely on the communication and cooperation of subunits. One of the emerging modalities for this underlying communication is bioelectricity [89], and future work will explore the mapping from the bioelectrical dynamics that implement neural-like [32] computations within cell networks to the robust plasticity observed with respect to dynamic form and function. A closely related set of questions concerns the implications of this computational property of all cells, not just neurons, for the material on which biological evolution acts [1].

For future directions, our multi-scale competency framework is easily extendable to simulate tissue growth via cell migration or division actions proposed by the underlying ANNs of the NCA. More specifically, our framework allows for a minimal set of biologically relevant uni-cellular actions, such as cell state update, cell division, migration, cell death, and an identity operation, only constrained by the NCA spatial grid. Furthermore, the framework is capable of handling flexible ANN architectures, potentially allowing us to investigate intriguing competencies, such as active inference [146], through utilizing world model architectures [147] in a (neuro)evolutionary context. Our system, so far, has a fixed hierarchical architecture that deviates from the scale-free competency architecture of biological life with open-ended functional adaptation (where any abstraction layer becomes the basis for the next one). Thus, in future work, we aim to model precisely this behavior by introducing multiple layers of horizontal communication pathways in an NCA that the ANN-based agents can dynamically traverse in the vertical direction. Moreover, by choosing a proper fitness function related to measuring scale-invariant pattern formation [107,148], critical dynamics [149,150,151,152,153,154], or applying the free-energy principle [146,155], we are confident that we will achieve a biologically more accurate model of the scale-free dynamics and open-ended evolution of life. Such computational models could thus further quantitative studies of the communication strategies and boundaries of individual and groups of cells in an agential, potentially adversarial umwelt, with possible applications in individual and collective aging (as morphostasis defects) [156,157], or cancer research [93,94,139].

## Figures and Tables

**Figure A1:**
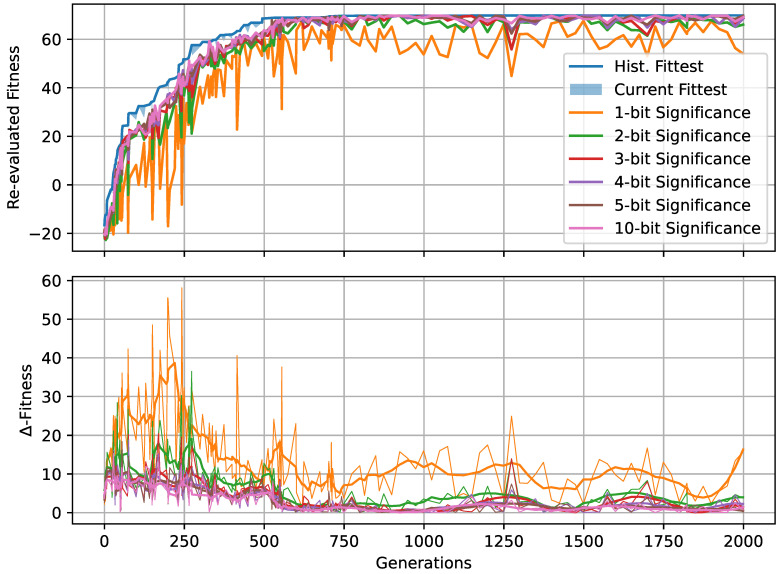
(**Top**) Re-evaluation of the fitness scores of the best-performing individual per generation of the lineage depicted in Figure 3 with modified genomes of systematically reduced floating-point precision by first type-casting from 32-bit (single-) to 16-bit (half-)precision and further reducing the number of significant bits (mantissa) in the genes’ binary representation from 10 to 1 (color-coded). Thus, for the 16-bit half-precision genes, the number of bits used to encode each gene is given by 6+S, with *S* significance (or mantissa) bits, 5 exponent bits, and 1 sign bit (following the IEEE 754-2008 standard). (**Bottom**) Similar to the top panel but showing the numerical difference of the historically best fitness score (blue curve in top panel) and the re-evaluated fitness scores (thin lines); thick lines on the bottom panel show the fitness deviation smoothed over several generations.

**Figure A2:**
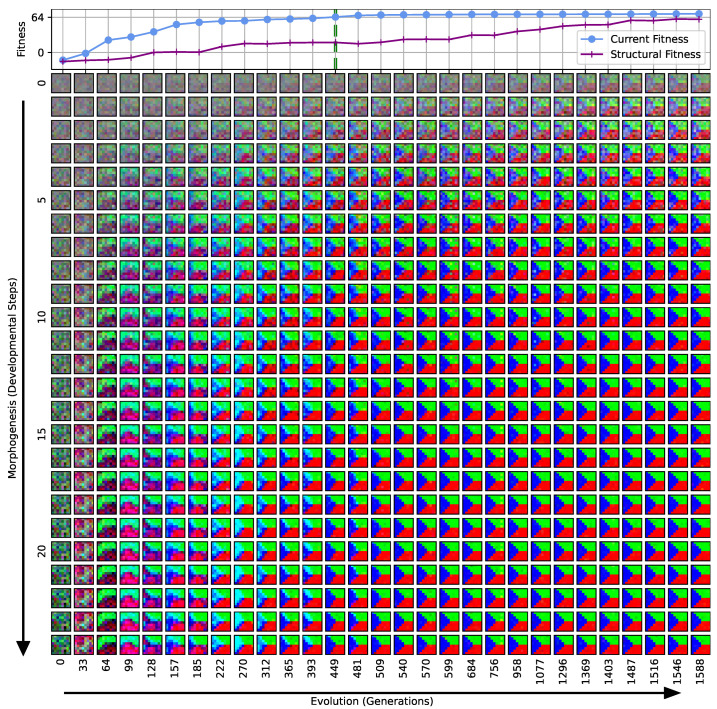
The developmental process of the 8×8 Czech flag task (vertical axis) of selected generations over evolutionary time scales (horizontal axis) for an NCA evolved with system parameters $\xi_{c}=0.25$, $P_{D}=50\%$, and R=4. Each pixel corresponds to a cell of the NCA, at a given developmental step and generation, in an RGB notation corresponding to the numerical values of the first three cell states, scaled to values between [0,1]. The top panel shows the current fitness of the respective generations (blue), and the structural fitness at $t_{k}=0$ (purple); the green vertical dashed line marks the generation crossing the fitness threshold of $F_{j}=64$, where we consider the problem solved.

**Figure A3:**
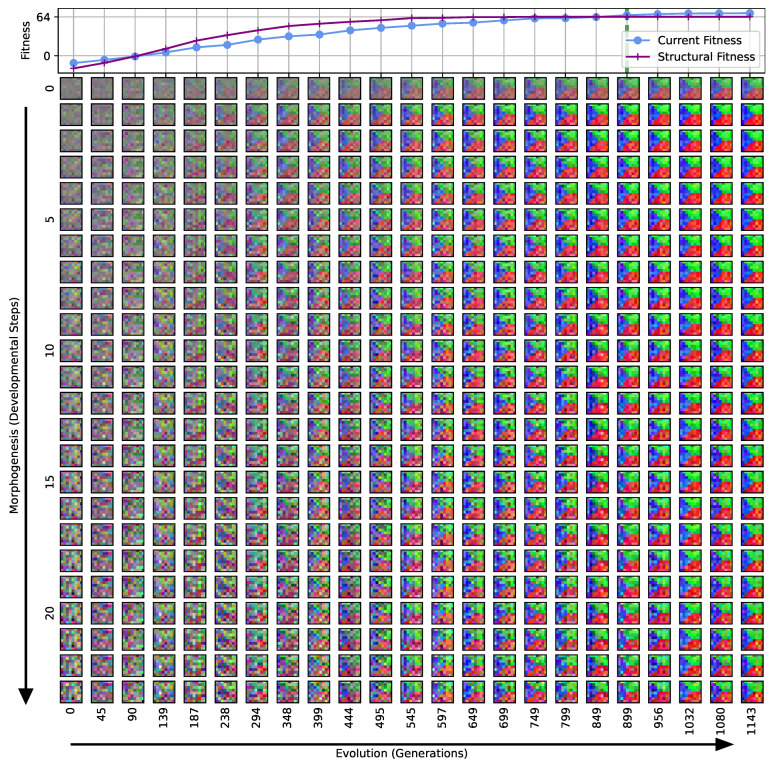
Same as Figure A2 but for an NCA without competency (i.e., $P_{D}=0$).

**Figure A4:**
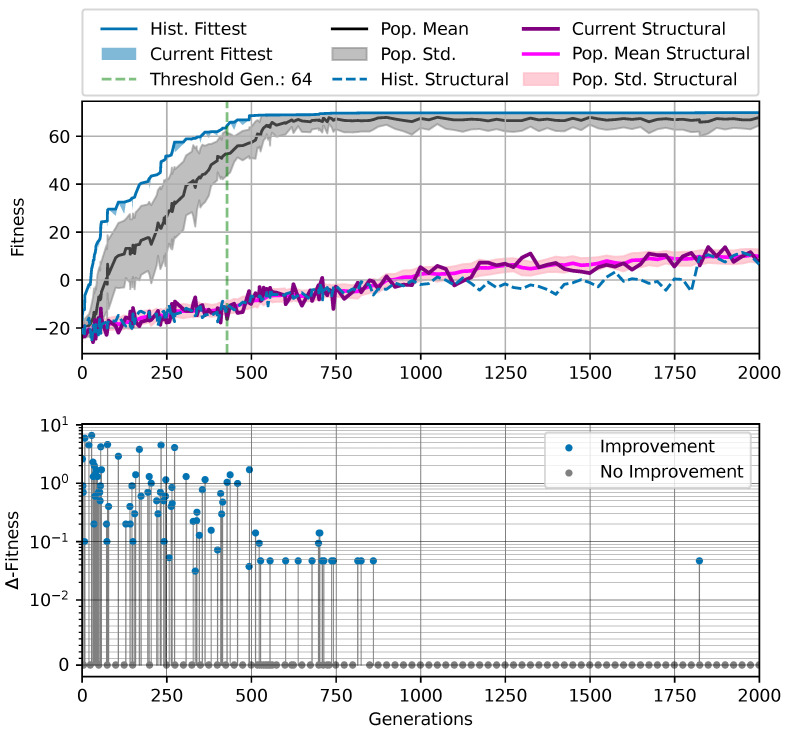
(**Top**) The same experimental data as shown in the bottom panel of Figure 3, additionally contrasting the structural fitness of the historically best-performing individual (dashed blue) with the current structural fitness (purple) that corresponds to the best-performing individual from the current generation. (**Bottom**) The numerical improvement in the maximum fitness score for every generation with respect to the historically best fitness score until the respective prior generation is drawn in a sym-log representation (linear scale between 0–$10^{-2}$, logarithmic above).

**Figure A5:**
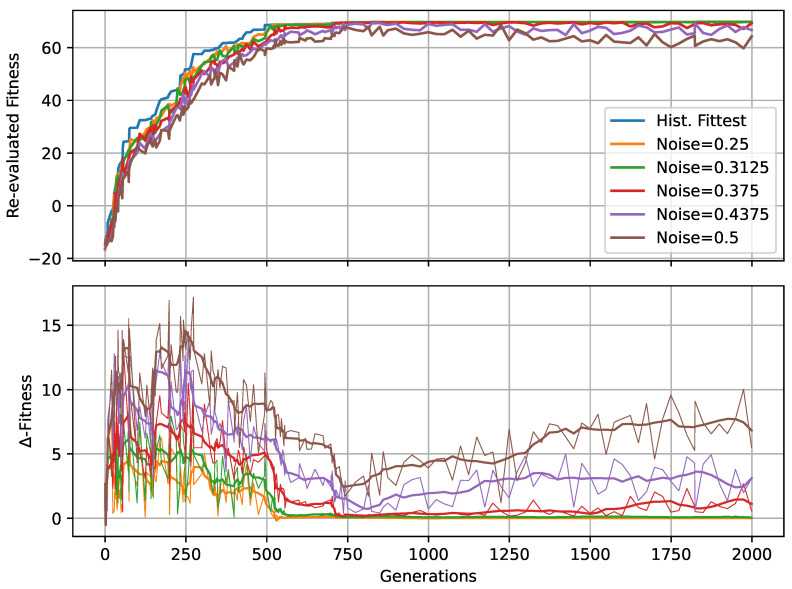
(**Top**) Re-evaluated fitness scores of the lineage depicted in the bottom panel of Figure 3 (and in Figure A4) at noise levels $\xi_{c}>0.25$, different from those originally experienced in the corresponding evolutionary process; the historically best fitness score (blue) is presented as reference. (**Bottom**) the numerical difference between the historically best fitness score and the fitness score of the best-performing individual of a particular generation (thin lines); thick lines in the bottom panel show the fitness deviation smoothed over several generations.

**Figure A6:**
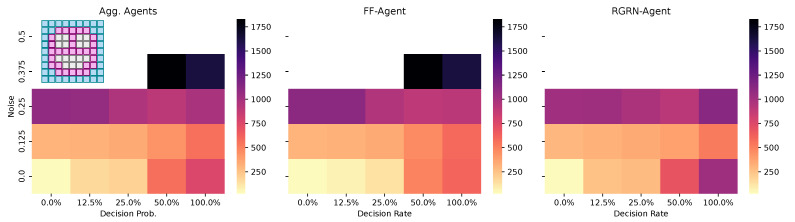
Same as Figure 4C but for a different target pattern, namely, a 9×9 smiley face (inset in left panel). Moreover, we here aggregate over R≥4.

**Figure A7:**
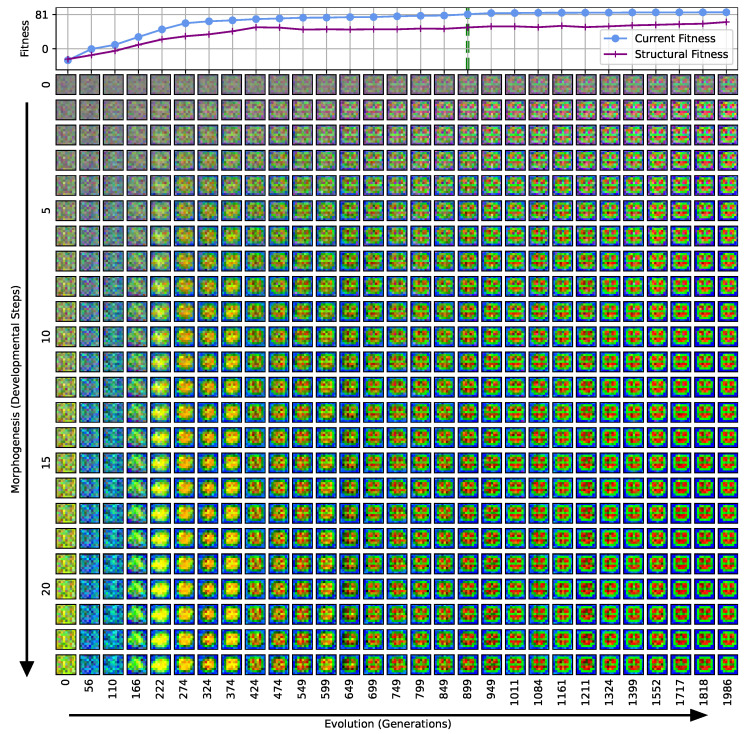
Same as Figure A2 but for the 9×9 smiley face task, with a fitness threshold of $F_{j}=81$ (cf. green vertical dashed line).

## Data Availability

Computational protocols and numerical data that support the findings of this study are shown in this article and in the Appendix A.

## Appendix A. Artificial Neural Networks

Inspired by biological neural circuits, an artificial neural network (ANN) is an interconnected network of artificial neurons (ANs) [158,159,160,161]. Each such AN maps a set of inputs $x\in R^{n}$ onto a single number y∈R, usually through a non-linear filter σ(.): the output of an AN can be defined as a parameterized function y=σ(w·x+b), with weights $w\in R^{n}$ and bias b∈R [162].

Commonly organized in layers of ANs, a *Feedforward* ANN represents a parameterized non-linear function $y^{\left(out\right)}=f_{\theta }^{\left(FF\right)}\left(x^{\left(1\right)}\right)$, transforming an input $x^{\left(1\right)}\in R^{N_{0}}$ over $i=1,\cdots ,N_{L}$ consecutive hidden layers of ANs $y^{\left(i\right)}\in R^{N_{i}}$ to an output vector $y^{\left(out\right)}\in R^{N_{L}}$. More specifically, the output $y^{\left(i\right)}\in R^{N_{i}}$ of layer *i* defined by

(A1) $$y^{\left(i\right)}=\sigma \left(W^{\left(i\right)}\cdot x^{\left(i\right)}+b^{\left(i\right)}\right)$$

becomes the input $x^{\left(i+1\right)}=y^{\left(i\right)}$ to the next *deeper* layer i+1 through layer-wise filtered dot products with the weight matrices $W^{\left(i\right)}=\left\{w_{jk}^{\left(i\right)}\right\}\in R^{N_{i}\times N_{i-1}}$ and bias vectors $b^{\left(i\right)}=\left(b_{1}^{\left(i\right)},\cdots ,b_{N_{i}}^{\left(i\right)}\right)\in R^{N_{i}}$.

Training an ANN thus boils down to optimizing a set of parameters $\theta =\{w_{jk}^{\left(i\right)},b_{k}^{\left(i\right)}\}$, i.e., the entire network’s weights and biases such that an input is mapped (with minimal deviation) to a desired output [113,163,164,165]. In this manuscript, we utilize ANNs as the trainable update function of a neural cellular automaton (NCA) [100] and optimize the corresponding ANN parameters via evolutionary algorithms to study the evolutionary implications of multi-scale intelligence on the example of morphogenesis.

Notably, in contrast to previous contributions of NCA-based morphogenesis [101], we do not rely on predefined convolutional filters in our ANN architectures to preprocess a cell’s local environment based on its own cell state $c_{i}\left(t_{k}\right)\equiv c_{i_{0}}\left(t_{k}\right)$ and the states of its $i_{\nu =1,\cdots ,N}$ direct neighbors $c_{i_{\nu }}\left(t_{k}\right)$ at a given time step $t_{k}$, which we formally collect in $N_{i}=\left(c_{i_{0}}\left(t_{k}\right),\cdots ,c_{i_{N}}\left(t_{k}\right)\right)$. Instead, we utilize a trainable *sensory* ANN $f_{\theta }^{\left(s\right)}\left(\cdot \right)$ that is applied individually to its own and every neighboring cell state to evaluate corresponding sensor embeddings $\varepsilon_{i_{\nu }}\left(t_{k}\right)=f_{\theta }^{\left(s\right)}\left(c_{i_{\nu }}\left(t_{k}\right)\right)\in R^{s}$. The sensor embedding vectors of a particular cell are averaged to form a cell-specific context vector $s_{i}\left(t_{k}\right)=\frac{1}{N+1}\left[\sum_{\nu =0}^{N}\varepsilon_{i_{\nu }}\left(t_{k}\right)\right]\in R^{s}$ of fixed size *s* that is permutation invariant with respect to the cell’s neighborhood on the NCA (also see Section 3.1). This context vector $s_{i}\left(t_{k}\right)$ represents the cell’s internal representation of its local environment on the cellular grid of the NCA.

Each cell *i* independently proposes an update $a_{i}\left(t_{k}\right)$ to its own state $c_{i}\left(t_{k}\right)$, potentially at every time step $t_{k}$ following Equation (1). This update is computed by a controller ANN $a_{i}\left(t_{k}\right)=f_{\theta }^{\left(c\right)}\left(s_{i}\left(t_{k}\right)\right)$, based on the cell-specific context vector $s_{i}\left(t_{k}\right)$. Thus, the set of ANN parameters of the NCA comprises the sensory and controller network parameters $f_{\theta }^{\left(s\right)}\left(\cdot \right)$ and $f_{\theta }^{\left(c\right)}\left(\cdot \right)$.

So far, we have not specified a particular architecture for either $f_{\theta }^{\left(s\right)}$ or $f_{\theta }^{\left(c\right)}$. Although the presented approach is agnostic to the particularly chosen ANN architecture, we here rely on rather simple implementations of ANNs: For the sensory ANN $f_{\theta }^{\left(s\right)}$, we utilize a *Feedforward* architecture with hyperbolic tangent activation function σ(·)=tanh(·) with four input units, eight neurons in a single hidden layer, and eight output neurons (defining the (s=8)-dimensional context vector), resulting in a total of 112 parameters. For the controller ANN, we utilize two different architectures, a *Feedforward* (cf. FF agent in Section 4.1 and Equation A1) and a recurrent ANN that is inspired by both *Recurrent* ANNs (RNNs) [108] and *Gene Regulatory Networks* [109] (cf. RGRN agent in Section 4.1 and Equations (A2) and (A3) below).

The *Feedforward* controller architecture consists of eight input units (i.e., the context vector from the sensory ANN), a single hidden layer with six neurons and a hyperbolic tangent activation function, and four output neurons without an activation function, resulting in 82 parameters in total; thus, the genuine *FF-agent* architecture in the main text comprises a total number of $N_{\mathrm{FF}}=194$ parameters (cf. Section 4.1). The *RGRN* controller architecture (see details below) consists of eight input units, a single self-regulated recurrent state with three neurons (with an internal hyperbolic tangent activation), and four output neurons (without an activation function), resulting in 52 parameters in total; thus the genuine *RGRN-agent* architecture in the main text comprises a total number of $N_{\mathrm{RGRN}}=164$ parameters (cf. Section 4.1). In Table A1, we summarize the FF-agent’s and RGRN-agent’s architectures and parameter counts.

**Table A1:** Architecture and number of parameters in sensory and controller ANNs of the two different agent architectures used in this contribution. The total number of parameters depends on the redundancy number *R* of the controller ANN (cf. Section 4.1).

	Sensory ANN (Num. Param.)	Controller ANN (Num. Param.)	Total Num. Param.
FF agent	Feedforward (112)	Feedforward (82)	112+R×82
RGRN agent	Feedforward (112)	RGRN (52)	112+R×52

Finally, we define the *RGRN* architecture $y\left(t_{k}\right)=f_{\theta }^{\left(RGRN\right)}\left(x\left(t_{k}\right),h\left(t_{k-1}\right)\right)$ that relies on both an instantaneous input $x\left(t_{k}\right)\in R^{I}$ and a recurrent state $h\left(t_{k-1}\right)\in R^{R}$ from the previous iteration of the network to generate an output $y\left(t_{k}\right)\in R^{O}$: first, we define the self-regulated recurrent state $h(t_{k})$ as

(A2) $$h\left(t_{k}\right)=\left(1-\tau_{1}\right)\times h\left(t_{k-1}\right)+\tau_{2}\times \left[\left(U\cdot x\left(t_{k}\right)+b_{U}\right)+\tanh\left(V\cdot h\left(t_{k-1}\right)+b_{V}\right)\right],$$

which is thus maintained over time by a factor of $\left(1-\tau_{1}\right)$ and updated by a factor of $\tau_{2}$ via integrating external stimuli $x(t_{k})$ and recurrent memory $h(t_{k-1})$ through the trainable matrices $U\in R^{H\times I}$ and $V\in R^{H\times H}$, and bias vectors $b_{U},b_{V}\in R^{H}$, respectively. Second, we evaluate the network’s output, $y\left(t_{k}\right)\in R^{O}$, based on the RGRN recurrent state $h(t_{k})$, following

(A3) $$y\left(t_{k}\right)=\sigma \left(W\cdot h\left(t_{k}\right)+b_{W}\right),$$

having introduced the weight matrix $W\in R^{O\times R}$ and bias vector $b_{W}\in R^{O}$, and a non-linear activation function σ(·).

Following ideas from Ref. [109], we thus utilize with Equation (A2) an ANN that maintains a self-regulated (or “gene regulated”) state $h(t_{k})$. However—and dropping the bias vectors for convenience below—the second term in Equation (A2), i.e., $\left[U\cdot x\left(t_{k}\right)+\tanh\left(V\cdot h\left(t_{k-1}\right)\right)\right]$, is reminiscent of the kernel of an RNN [108], thus allowing the RGRN to integrate new information (i.e., external stimuli) into its regulatory behavior. Thus, the state update of $h(t_{k})$ corresponds to regulating the network’s recurrent state (or “gene expression”) conditional to external stimuli. Furthermore, explicitly separating the self-regulated recurrent state from the RGRN output allows us to utilize the RGRN as a controller, i.e., to use its output $y(t_{k})$ for updating the cell state of an NCA in Equation (1).

Here, we set $\tau_{1}=0.75$, $\tau_{2}=0.25$ and choose σ(·) as the identity transformation (i.e., no or linear activation of the RGRN output), and we apply Equation (A2) three times (updating $h(t_{k})$ in every cycle) before forwarding the final value of $h(t_{k})$ to Equation (A3) to generate the RGRN output.

For all ANN implementations, we here rely on PyTorch [166].

## Appendix B. A Reinforcement Learning Agent’s Perception–Action Cycle

We utilize a Neural Cellular Automaton (NCA) [100] for morphogenesis tasks of two-dimensional target patterns. In such a setting, each cell of the NCA represents an autonomous agent that perceives details about its local environment (i.e., the cell states of its direct neighbors on the NCA spatial grid) and proposes actions to update its own state. Here, we summarize the terminology behind this perception–action cycle of an agent in an arbitrary environment of a Reinforcement Learning (RL) setting [113] (see Figure 2A for an illustration).

Based on an agent’s perception of the environment, i.e., a state $s_{k}$ measured at time step $t_{k}$, the agent’s goal is to manipulate the environment by taking an action $a_{k}$—resulting in a state update $s_{k+1}$ in the next time step—to collect as much reward $r_{k}\in R$ (provided by the environment) as possible.

Formally, an agent picks its actions according to a policy $\pi_{\theta }\left(s_{k^{\prime }\leq k}\right)\to a_{k}$, i.e., a typically complicated function which might be parameterized via hidden variables θ. Artificial neural networks (ANNs) as universal function approximators [112] are promising candidates that can be *trained* to *fit* an agent’s optimal policy (mapping states $s_{k}$ to optimal actions $a_{k}$ [113]). Here, we thus utilize ANNs as a trainable update function of an NCA and deploy evolutionary algorithms (see Section 3.2) to find the optimal policy (here, of morphogenesis tasks) $\pi_{\theta^{*}}\left(s_{k^{\prime }\leq k}\right)$ via optimizing $\theta^{*}=\mathrm{argma}x_{\theta }\left(\sum_{k^{\prime }\leq k}r_{k^{\prime }}\right)$. This enables an agent to choose actions aiming at maximizing the expected cumulative reward (or maximum fitness, in our terms).

The particular functional choice of the reward signal defines the agent’s task via positive (or negative) reinforcement. In our case, the cumulative reward $R_{a}$ of all $N_{a}$ agents (i.e., of all cells on the grid) after $t_{D}$ time steps is summed up to the fitness $f=\sum R_{a}$ of the entire NCA. There is no general procedure for creating effective reward signals.

Crucially, we here do not provide the cellular agents with environmental reward feedback directly but only use the cumulative reward $R_{a}$ as a fitness criterion for the evolutionary algorithm. Thus, the collective of cells needs to evolve a signaling strategy to communicate desirable or prohibitive cell state updates during the corresponding developmental stage.

## Appendix C. Fixed Boundary Condition Handling of the Neural Cellular Automaton

We employ neural cellular automata (NCAs) with fixed boundary conditions on a two-dimensional square grid (see Section 3.1). Each cell *i* is associated with integer grid-coordinates $\left(x_{i},y_{i}\right)$ on the NCA grid of size $N_{x}\times N_{y}$, with $x_{i}\in \left[1,N_{x}\right]$ and $y_{i}\in \left[1,N_{y}\right]$.

The neighborhood of cell *i* is defined by all directly adjacent cells $i_{\nu =1,\cdots ,N}$, i.e., that share a border or a corner with cell *i*. Since we consider a square grid in this contribution, the grid coordinates of all N=8 neighbor cells $\left(x_{i_{\nu }},y_{i_{\nu }}\right)$ are given by the permutations of $\left(x_{i}\pm m,y_{i}\pm n\right)$ with m,n∈{0,1}, excluding the identity m=n=0.

For cells at the boundaries of the grid, some neighbors with coordinates $x_{i_{\nu }},y_{i_{\nu }}<1$ or $>N_{x},N_{y}$, respectively, will be out of bounds. Thus, we clip all neighbor coordinates to the intervals $\left[1,N_{x}\right]$ and $\left[1,N_{y}\right]$, respectively, via $x_{i_{\nu }}\to x_{i_{\nu }^{\prime }}=\min\left(\max\left(x_{i_{\nu }},1\right),N_{x}\right)$, and $y_{i_{\nu }}\to y_{i_{\nu }^{\prime }}=\min\left(\max\left(y_{i_{\nu }},1\right),N_{y}\right)$, and replace the neighbor index $i_{\nu }$ with the corresponding index $i_{\nu }^{\prime }$ of the respectively clipped coordinates $\left(x_{i_{\nu }^{\prime }},y_{i_{\nu }^{\prime }}\right)$. Collecting the numerical state values of the neighborhood of cell *i* thus yields $N_{i}\left(t_{k}\right)\to N_{i}^{\prime }\left(t_{k}\right)=\left(c_{i}\left(t_{k}\right),c_{i_{1}^{\prime }}\left(t_{k}\right),\cdots ,c_{i_{N}^{\prime }}\left(t_{k}\right)\right)$. The matrix $N_{i}^{\prime }\left(t_{k}\right)$ then represents the input of the sensory part of the NCA artificial neural network (cf. Section 3.1).

## Appendix D. Covariance Matrix Adaptation Evolutionary Strategy (CMA-ES)

The *Covariance Matrix Adaptation Evolutionary Strategy* (CMA-ES) [103] is a popular evolutionary algorithm: a multivariant normal distribution is utilized to model the (genotypic-) distribution of a set or a population of parameters that are evaluated against an objective function. Roughly speaking, this evaluated fitness score of an individual is associated with its probability of survival and thus for participating in the reproduction of the next generation. The parameters of the multivariant normal distribution, i.e., the mean and covariance matrix, are successively updated based on selecting the best individuals from a given population (or, more precisely, by weighting the relative importance of an individual by its fitness score) such that high-fitness individuals are generated with high likelihood by the Gaussian model. Thus, iteratively sampling “offspring” generations and adapting the model covariance matrix (and its mean) based on the population’s fitness scores guides the evolutionary population toward high-fitness regions in the parameter space over successive generations. Typically, also the numerical step size of the parameter update is adapted according to some inter- and intra-generation fitness measures. In a nutshell [103]:

1. CMA-ES typically starts with a standard (or parameterized) multi-variant normal distribution with the dimension given by the number of parameters (or genes).
2. At each evolutionary cycle, a new population of a fixed number of individuals is sampled from the model.
3. Each individual is evaluated against a fitness function, which quantifies the corresponding individual’s probability of being selected for reproduction to form the next generation.
4. The mean and covariance matrix of the normal distribution, and a step-size parameter, are updated such that high-quality individuals are generated with high likelihood by the generative model.
5. The process (2–5) is repeated until a convergence criterion is met.

In the CMA-ES experiments presented in this contribution, we use an initial normal distribution with zero mean $\mu_{0}=0$ and a standard deviation of typically $\sigma_{0}=2^{-4}$, and we disable step-size adaptation.

We specifically utilize the open-source *pycma* Python implementation of CMA-ES from Ref. [167]. CMA-ES utilizes floating point numbers as genes. Since we rely on the PyTorch [166] framework to encode both structural and functional genes, we use single-precision (32-bit) floating point numbers per default during training. Here, we study the effects of varying the bit-precision encodings of the genes on the performance of an evolving NCA. Relying on genetic data of the entire lineage depicted in Figure 3, we systematically reduce the number of significance bits of the best-performing individual for every generation, and re-evaluate the corresponding fitness scores with the altered genome. The results depicted in Figure A1 indicate that as evolution progresses, the bit precision of the genes of high-quality individuals can effectively be reduced by a factor of four, rendering our approach numerically robust: while a reduced 7-bit encoding leads to significant deviations in the re-evaluated fitness score also after convergence of ≈10% (with minimal deviations around generation 750–860, cf. Appendix F), a reduced 8-bit encoding only induces deviations of ≈1% in the converged fitness scores, and ≥9-bit genes virtually encode the same behavior as their single-precision genes (here, after generation 860, cf. Appendix F).

## Appendix E. Direct vs. Multi-Scale Encoding: Morphogenetic Development over Evolutionary Time Scales

In Figure A2, we explicitly illustrate the developmental process of the 8×8 Czech flag task over evolutionary time scales for an NCA evolved at the noise level of $\xi_{c}=0.25$, decision-making probability $P_{D}=50\%$, and redundancy number R=4; in Figure A3, we illustrate the same developmental process for an NCA without competency, i.e., with $P_{D}=0$.

These two figures illustrate how the evolutionary process learns how to construct the target pattern over generations, depending on the competency of the underlying substrate: either driven by intercellular communication-based self-assembly of the target pattern that continuously corrects potential errors of the developmental program, or via directly encoding the target pattern into the structural part of the genome to resist developmental noise for as long as possible. Moreover, while the target pattern for the competent first case is quickly (and robustly) self-assembled and maintained over time—potentially much longer as the $t_{D}=25$ developmental time steps the phenotypes have been selected for—in the direct case, the initial cell state is eventually destroyed by the noise during the developmental process.

Thus, in the former case, illustrated in Figure A2, the structural fitness of the initial cell states (at $t_{k}=0$) remains decoupled and rather low compared to the highest fitness of the population of the phenotypes, even long after the problem is solved. In contrast, in the latter case, illustrated in Figure A3, the initial cell state needs to evolve towards the target pattern directly, resulting in high structural fitness values at $t_{k}=0$, which are then progressively decreased by the noise during the developmental process, resulting in correspondingly lower phenotypic fitness values.

## Appendix F. Direct vs. Multi-scale Encoding: Neutral Transfer of Hierarchical Competencies Affects Uni-Cellular Robustness

In Section 4.2, Figure 3, we compare an evolutionary process operating on (i) the direct encoding of the structural traits of an 8×8 Czech flag pattern with the evolution of (ii) the parameters of an NCA-based multi-scale encoding of the same pattern. Here, we specifically investigate case (ii) more closely, relating the slow but steady long-term improvement in the structural fitness, long after the problem is effectively converged, to the Baldwin effect [14], and neutral evolution and the “paradox of robustness” [51,52,53,121,122,123,124,125,126,127].

In the top panel of Figure A4, we again show the entire evolutionary lineage presented in the bottom panel of Figure 3, but we specifically contrast the structural fitness of the historically best-performing individual with the structural fitness of the best-performing individual of every generation. We see that after generation 860, the historically best structural fitness (which we explicitly track for numerical reasons) deviates from the structural fitness of the still-evolving population.

We interpret this slow but steady improvement in the structural fitness as a manifestation of the Baldwin effect [14], where (evolutionarily) acquired uni-cellular competencies—here, to assemble a target pattern—are shifted to hard-wired phenotypical traits. Especially between generations 860 and 1823, this transfer of competency is caused by corresponding successive adaptations to the structural genomes of the entire population as reflected by the increasing mean of the entire population’s structural fitness depicted in Figure A4. However, this process happens without significant changes to the entire system’s fitness score between successive generations as illustrated in the bottom panel of Figure A4, where we explicitly present incremental numerical improvements to the lineage’s fitness score. Thus, these adaptations to the structural genomes remain neutral with respect to the fitness of the entire system.

Eventually, a random event in generation 1823 causes a slight improvement in the so-far historically best fitness score of generation 860 (from f=69.781 to 69.828). However, this newly assigned historically best individual is now qualitatively different from the previous one (cf. purple and blue-dashed curves in Figure A4), as its policy is shifted from a rather competency-based NCA (generations 860–1822) to a solution that relies more heavily on directly encoded phenotypic traits (generations ≥1823). This, in turn, affects the new solution’s robustness against increasingly noisy cell state updates as illustrated in Figure A5: we re-evaluate the fitness score of the entire genetic lineage depicted in Figure A4 (which we tracked in the original experiment) at modified noise levels larger than experienced during the respective evolutionary process $\xi_{c}>0.25$. While the best-performing individuals for generations 750–860 can generalize well to different noise levels, we observe successively decreasing performance of the preceding generations, especially at higher noise levels.

Thus, this could be a manifestation of the paradox of robustness, which states [51,121] that in the presence of a higher-level control mechanism, the system’s lower-level (agential) components may become increasingly unreliable. In our case, the higher-level mechanism would be represented by an increasingly accurate initial cell state configuration, which is a global system-level encoding of the target pattern. The lower-level components are represented by the ANN-based uni-cellular agents of the NCA, performing local error correction to assemble and maintain the target pattern through cell state updates. Notably, and depending on the noise level, the former may be more difficult to acquire by an evolutionary process, starting from a randomly initialized population, but the latter might become increasingly specialized the better the structural genome is adapted.

Since for a particular noise level, a precise enough initial cell state configuration is sufficient to solve the problem (cf. Figure 6), the NCA competencies may in such cases become successively unnecessary in the long run, and thus lose their relevance to the evolutionary process. Consequently, such a shift in competency renders a system less robust against perturbations and changing environmental conditions as depicted in Figure A5 (and also reflected in Figure A1). Notably, such a combination of the Baldwin effect (shift in competencies), neutral evolution (not affecting the system-level fitness scores), and the paradox of robustness (less competent components) might even explain the reduced capabilities of NCAs pre-evolved at lower rather than higher noise levels to adapt to modified problems of self-assembling different target patterns as observed in Figure 7.

We also conjecture that this mechanism of heterogeneous agents’ competencies cooperating on a common system-level objective might be important for adaptability and evolvability in biological systems. Notably, robustness mechanisms might be only partially active. For example, chaperones that prevent proteins from misfolding allow for more exploration of mutations while destabilizing the fold, giving the system more time to find a restabilizing mutation (see Ref. [168]). In such cases, the "protected layers" are still intermittently exposed to selection and unlikely to deteriorate. However, if the specialized competencies of certain agential components can be replaced by a different mechanism in the same organism more cheaply, these increasingly redundant components might be repurposed to fulfill different tasks, thus potentially facilitating open-ended evolution.

## Appendix G. Direct vs. Multi-Scale Encoding: Evolution and Morphogenesis of a Smiley Face Pattern

In the main text, we primarily investigate the evolutionary implications of multi-scale intelligence on the example of morphogenesis of an 8×8 Czech flag pattern. To test whether our findings in Section 4.3 generalize to different target patterns, we here present results for a much more involved task, namely, a 9×9 smiley face pattern (cf. Figure 1), which has several internal boundaries of (i) the face, (ii) the eyes, and (iii) the mouth; all other parameters are the same as for the 8×8 Czech flag task.

We thus perform an analogous study to Section 4.3, and present the results in Figure A6 (reminiscent of Figure 4C) but for redundancy numbers R≥4 (as we found that smaller controller networks perform systematically worse on the task, suggesting a capacity bottleneck of ANNs with R<4 in this case). Analogously to the much simpler 8×8 Czech task, we can learn from Figure A6 that, while in the low-noise regime, direct encoding can lead to a more efficient evolutionary process, in situations with increasing developmental noise, higher competency levels (here again realized via the decision-making probability) can significantly enhance the efficiency of the evolutionary process of a morphogenesis task. Notably, and due to computational reasons, we evaluate only two to three independent evolutionary processes for every combination of the system parameters (noise-level, decision-making probability, and redundancy number) for the results depicted in Figure A6. However, the overall trend of the evolutionary efficiency of (i) directly encoding the target pattern and (ii) encoding the functional parameters of a multi-scale competency architecture is consistent with our previous results discussed in Section 4.3. Due to the increased complexity of the 9×9 smiley face task, the critical noise level that separates the evolutionary efficiency of (i) and (ii) is correspondingly shifted to larger values of, here, $\xi_{c}\geq 0.25$ (cf. Figure 4C).

Analogous to Figure A2, we illustrate in Figure A7 the developmental process of the 9×9 smiley face task over evolutionary time scales for an NCA evolved at the noise level of $\xi_{c}=0.25$, decision-making probability $P_{D}=50\%$, and redundancy number of R=4 in an RGB scheme attributing the numerical values of the first three cell states, scaled to values between [0,1], respectively (cf. Section 3.1).

## Appendix H. Evolution Exploits Redundancy at the Cost of a More Complex Search Space

Analogous to Section 4.3, we here present the evolutionary efficiency of the same morphogenesis experiments of the 8×8 Czech flag problem depicted in Figure 4 but present as a function of the redundancy number *R*—instead of the decision-making probability $P_{D}$—and the noise level $\xi_{c}$; for a given combination of *R* and $\xi_{c}$, we additionally utilize different values for $P_{D}=\left\{0.25,0.5,1.0\right\}$ and perform 15 statistically independent runs of the EA for each parameter combination, resulting in 45 independent evolutionary runs per (*R*, $\xi_{c}$)-tuple.

Although there appears to be an effect of *R* on the evolutionary efficiency (cf. Figure A8A,C,D), the results are less pronounced compared to Figure A8. Despite considerable uncertainty in the evolutionary efficiency as shown in Figure A8A,B, we can learn from the heatmaps, Figure A8C,D, that at low noise levels of $\xi_{c}=0$ or 0.125, large *R* values appear favorable over lower ones, whereas, at larger noise levels of $\xi_{c}=0.5$, populations with lower values of *R* perform better on average. For intermediate noise levels of $\xi_{c}=0.25$ and 0.375, we observe an “optimal” redundancy number of 4 in this particular example.

This suggests a trade-off in the evolutionary efficiency of redundancy—as an affordance of competency—and the corresponding increase in the number of overall parameters of the functional genome.

## Appendix I. Morphogenesis at Scale with a Hybrid Compositional Pattern-Producing Network—Neural Cellular Automata Model

The particular choice of especially the structural part of the genome $x_{j}^{\left(S\right)}$ in Equation (3) limits the scalability of our multi-scale competency approach of morphogenesis to significantly larger systems, as the size of the structural genome will grow correspondingly with the number of cells in the system. However, by utilizing Compositional Pattern Producing Networks (CPPNs) [120,141], the parameters $\theta_{H}$ of a hyper-network $f_{\theta }^{\left(H\right)}\left(\cdot \right)$ could replace the structural genes in Equation (3) such that the initial states of each cell *i* are indirectly encoded by the hyper-network based on their relative spatial positions $\left(x_{i}/N_{x},y_{i}/N_{y}\right)$ on the grid of the Neural Cellular Automaton (NCA) via $c_{i}\left(0\right)=f_{\theta }^{\left(H\right)}\left(x_{i}/N_{x},y_{i}/N_{y}\right)$.

However, it has proven to be difficult, if not numerically infeasible, to reliably and exactly reproduce a two-dimensional target pattern using CPPNs [142]. Thus, we here propose a hybrid approach for morphogenesis at the scale of a CPPN, indirectly encoding the initial cell states of an NCA, whose uni-cellular agents are then challenged to self-assemble the desired target pattern in a morphogenetic developmental stage. This would allow for scaling the target pattern arbitrarily either during training or during deployment since the number of cells on the NCA grid does not affect the size of the (structural part of the) genome.

## References

[1] Levin M. (2023). Darwin’s agential materials: Evolutionary implications of multiscale competency in developmental biology. Cell. Mol. Life Sci..

[2] Fields C., Levin M. (2023). Regulative development as a model for origin of life and artificial life studies. Biosystems.

[3] Fields C., Levin M. (2022). Competency in Navigating Arbitrary Spaces as an Invariant for Analyzing Cognition in Diverse Embodiments. Entropy.

[4] Levin M. (2023). Collective Intelligence of Morphogenesis as a Teleonomic Process. Evolution “On Purpose”: Teleonomy in Living Systems.

[5] Birnbaum K.D., Alvarado A.S. (2008). Slicing across Kingdoms: Regeneration in Plants and Animals. Cell.

[6] Harris A.K. (2018). The need for a concept of shape homeostasis. Biosystems.

[7] Levin M., Pietak A.M., Bischof J. (2019). Planarian regeneration as a model of anatomical homeostasis: Recent progress in biophysical and computational approaches. Semin. Cell Dev. Biol..

[8] Vandenberg L.N., Adams D.S., Levin M. (2012). Normalized shape and location of perturbed craniofacial structures in the Xenopus tadpole reveal an innate ability to achieve correct morphology. Dev. Dyn..

[9] Cooke J. (1981). Scale of body pattern adjusts to available cell number in amphibian embryos. Nature.

[10] Fankhauser G. (1945). Maintenance of normal structure in heteroploid salamander larvae, through compensation of changes in cell size by adjustment of cell number and cell shape. J. Exp. Zool..

[11] Blackiston D.J., Levin M. (2013). Ectopic eyes outside the head in Xenopus tadpoles provide sensory data for light-mediated learning. J. Exp. Biol..

[12] Pezzulo G., Levin M. (2015). Re-membering the body: Applications of computational neuroscience to the top-down control of regeneration of limbs and other complex organs. Integr. Biol..

[13] Davies J., Levin M. (2023). Synthetic morphology with agential materials. Nat. Rev. Bioeng..

[14] Baldwin J.M. (1896). A New Factor in Evolution. Am. Nat..

[15] Thorpe W.H. (1945). Animal Learning and Evolution. Nature.

[16] Simpson G.G. (1953). The Baldwin Effect. Evolution.

[17] Hinton G.E., Nowlan S.J. (1987). How learning can guide evolution. Complex Syst..

[18] Belew R.K. When Both Individuals and Populations Search: Adding Simple Learning to the Genetic Algorithm. Proceedings of the 3rd International Conference on Genetic Algorithms.

[19] Ackley D., Littman M. (1991). Interactions between learning and evolution. Artif. Life II.

[20] Mayley G. (1996). Landscapes, Learning Costs, and Genetic Assimilation. Evol. Comput..

[21] Bull L. (1999). On the Baldwin Effect. Artif. Life.

[22] Nolfi S., Floreano D. (1999). Learning and Evolution. Auton. Robot..

[23] Dopazo H. (2001). A Model for the Interaction of Learning and Evolution. Bull. Math. Biol..

[24] Weber B.H., Depew D.J. (2003). Evolution and Learning: The Baldwin Effect Reconsidered.

[25] Mery F., Kawecki T.J. (2003). A fitness cost of learning ability in *Drosophila melanogaster*. Proc. R. Soc. Lond. Ser. B Biol. Sci..

[26] Crispo E. (2007). The Baldwin effect and genetic assimilation: Revisiting two mechanisms of evolutionary change mediated by phenotypic plasticity. Evolution.

[27] Kawecki T.J. (2009). Evolutionary ecology of learning: Insights from fruit flies. Popul. Ecol..

[28] Paenke I., Kawecki T.J., Sendhoff B. (2009). The Influence of Learning on Evolution: A Mathematical Framework. Artif. Life.

[29] Hoedjes K.M., Kruidhof H.M., Huigens M.E., Dicke M., Vet L.E.M., Smid H.M. (2010). Natural variation in learning rate and memory dynamics in parasitoid wasps: Opportunities for converging ecology and neuroscience. Proc. R. Soc. B Biol. Sci..

[30] Watson R.A., Szathmáry E. (2016). How Can Evolution Learn?. Trends Ecol. Evol..

[31] Livnat A., Papadimitriou C. (2016). Evolution and Learning: Used Together, Fused Together. A Response to Watson and Szathmáry. Trends Ecol. Evol..

[32] Fields C., Bischof J., Levin M. (2020). Morphological Coordination: A Common Ancestral Function Unifying Neural and Non-Neural Signaling. Physiology.

[33] Baluška F., Mancuso S. (2009). Deep evolutionary origins of neurobiology: Turning the essence of ’neural’ upside-down. Commun. Integr. Biol..

[34] Baluška F., Miller W.B., Reber A.S. (2022). Cellular and evolutionary perspectives on organismal cognition: From unicellular to multicellular organisms. Biol. J. Linn. Soc..

[35] Shreesha L., Levin M. (2023). Cellular Competency during Development Alters Evolutionary Dynamics in an Artificial Embryogeny Model. Entropy.

[36] Liard V., Parsons D.P., Rouzaud-Cornabas J., Beslon G. (2020). The Complexity Ratchet: Stronger than Selection, Stronger than Evolvability, Weaker than Robustness. Artif. Life.

[37] Huizinga J., Stanley K.O., Clune J. (2018). The Emergence of Canalization and Evolvability in an Open-Ended, Interactive Evolutionary System. Artif. Life.

[38] Ratcliff W.C., Fankhauser J.D., Rogers D.W., Greig D., Travisano M. (2015). Origins of multicellular evolvability in snowflake yeast. Nat. Commun..

[39] Payne J.L., Moore J.H., Wagner A. (2014). Robustness, Evolvability, and the Logic of Genetic Regulation. Artif. Life.

[40] Raman K., Wagner A. (2011). The evolvability of programmable hardware. J. R. Soc. Interface.

[41] Nehaniv C.L. (2003). Evolvability. Biosystems.

[42] Hoenigsberg H. (2003). Cell biology, molecular embryology, Lamarckian and Darwinian selection as evolvability. Genet. Mol. Res..

[43] Hansen T.F. (2003). Is modularity necessary for evolvability?. Biosystems.

[44] Bedau M.A., Packard N.H. (2003). Evolution of evolvability via adaptation of mutation rates. Biosystems.

[45] Kirschner M., Gerhart J. (1998). Evolvability. Proc. Natl. Acad. Sci. USA.

[46] Wagner G.P., Altenberg L. (1996). Perspective: Complex Adaptations and the Evolution of Evolvability. Evolution.

[47] Raff R. (1994). Developmental mechanisms in the evolution of animal form: Origins and evolvability of body plans. Early Life Earth.

[48] Raff R. (1996). The Shape of Life: Genes, Development, and the Evolution of Animal Form.

[49] Alberch P. (1991). From genes to phenotype: Dynamical systems and evolvability. Genetica.

[50] Frank S.A. (2009). Natural selection maximizes Fisher information. J. Evol. Biol..

[51] Frank S.A. (2007). Maladaptation and the Paradox of Robustness in Evolution. PLoS ONE.

[52] Frank S.A. (1997). Developmental selection and self-organization. Biosystems.

[53] Frank S.A. (1997). The Design of Adaptive Systems: Optimal Parameters for Variation and Selection in Learning and Development. J. Theor. Biol..

[54] Stanley K.O., Miikkulainen R. (2003). A Taxonomy for Artificial Embryogeny. Artif. Life.

[55] Waddington C. (1957). The Strategy of the Genes.

[56] Waddington C.H. (2014). The Strategy of the Genes.

[57] Noble D. (2022). Modern physiology vindicates Darwin’s dream. Exp. Physiol..

[58] Shapiro J.A. (2022). Engines of innovation: Biological origins of genome evolution. Biol. J. Linn. Soc..

[59] Szilágyi A., Szabó P., Santos M., Szathmáry E. (2020). Phenotypes to remember: Evolutionary developmental memory capacity and robustness. PLoS Comput. Biol..

[60] Szathmáry E. (2015). Toward major evolutionary transitions theory 2.0. Proc. Natl. Acad. Sci. USA.

[61] Watson R.A., Levin M., Buckley C.L. (2022). Design for an Individual: Connectionist Approaches to the Evolutionary Transitions in Individuality. Front. Ecol. Evol..

[62] Kouvaris K., Clune J., Kounios L., Brede M., Watson R.A. (2017). How evolution learns to generalise: Using the principles of learning theory to understand the evolution of developmental organisation. PLoS Comput. Biol..

[63] Fox Keller E. (1999). Elusive locus of control in biological development: Genetic versus developmental programs. J. Exp. Zool..

[64] Jablonka E. (2017). The evolutionary implications of epigenetic inheritance. Interface Focus.

[65] Laland K., Uller T., Feldman M., Sterelny K., Müller G.B., Moczek A., Jablonka E., Odling-Smee J., Wray G.A., Hoekstra H.E. (2014). Does evolutionary theory need a rethink?. Nature.

[66] Elgart M., Snir O., Soen Y. (2015). Stress-mediated tuning of developmental robustness and plasticity in flies. Biochim. Biophys. Acta (BBA) Gene Regul. Mech..

[67] Eldakar O.T., Wilson D.S. (2011). Eight Criticisms not to make about Group Selection. Evolution.

[68] Karve S., Wagner A. (2022). Environmental complexity is more important than mutation in driving the evolution of latent novel traits in *E. coli*. Nat. Commun..

[69] Karve S., Wagner A. (2022). Multiple novel traits without immediate benefits originate in bacteria evolving on single antibiotics. Mol. Biol. Evol..

[70] Wagner A. (2011). The molecular origins of evolutionary innovations. Trends Genet..

[71] Wagner A., Rosen W. (2014). Spaces of the possible: Universal Darwinism and the wall between technological and biological innovation. J. R. Soc. Interface.

[72] Wilson D.S. (1975). A theory of group selection. Proc. Natl. Acad. Sci. USA.

[73] Calabretta R., Ferdinando A.D., Wagner G.P., Parisi D. (2003). What does it take to evolve behaviorally complex organisms?. Biosystems.

[74] Schlosser G., Wagner G.P. (2004). Modularity in Development and Evolution.

[75] Stadler B.M., Stadler P.F., Wagner G.P., Fontana W. (2001). The Topology of the Possible: Formal Spaces Underlying Patterns of Evolutionary Change. J. Theor. Biol..

[76] Ten Tusscher K.H., Hogeweg P. (2011). Evolution of Networks for Body Plan Patterning; Interplay of Modularity, Robustness and Evolvability. PLoS Comput. Biol..

[77] Wagner G.P., Pavlicev M., Cheverud J.M. (2007). The road to modularity. Nat. Rev. Genet..

[78] Wagner G.P., Stadler P.F. (2003). Quasi-Independence, Homology and the Unity of Type: A Topological Theory of Characters. J. Theor. Biol..

[79] Power D.A., Watson R.A., Szathmáry E., Mills R., Powers S.T., Doncaster C.P., Czapp B. (2015). What can ecosystems learn? Expanding evolutionary ecology with learning theory. Biol. Direct.

[80] Schreier H.I., Soen Y., Brenner N. (2017). Exploratory adaptation in large random networks. Nat. Commun..

[81] Soen Y., Knafo M., Elgart M. (2015). A principle of organization which facilitates broad Lamarckian-like adaptations by improvisation. Biol. Direct.

[82] Uller T., Moczek A.P., Watson R.A., Brakefield P.M., Laland K.N. (2018). Developmental Bias and Evolution: A Regulatory Network Perspective. Genetics.

[83] Watson R.A., Mills R., Buckley C.L. (2011). Global Adaptation in Networks of Selfish Components: Emergent Associative Memory at the System Scale. Artif. Life.

[84] Watson R.A., Mills R., Buckley C.L., Kouvaris K., Jackson A., Powers S.T., Cox C., Tudge S., Davies A., Kounios L. (2016). Evolutionary Connectionism: Algorithmic Principles Underlying the Evolution of Biological Organisation in Evo-Devo, Evo-Eco and Evolutionary Transitions. Evol. Biol..

[85] Watson R.A., Wagner G.P., Pavlicev M., Weinreich D.M., Mills R. (2014). The evolution of phenotypic correlations and “developmental memory”. Evolution.

[86] Bongard J. (2011). Morphological change in machines accelerates the evolution of robust behavior. Proc. Natl. Acad. Sci. USA.

[87] Bongard J., Laschi C., Lipson H., Cheney N., Corucci F. (2016). Material properties affect evolution’s ability to exploit morphological computation in growing soft-bodied creatures. Artificial Life Conference Proceedings.

[88] Kriegman S., Cheney N., Bongard J. (2018). How morphological development can guide evolution. Sci. Rep..

[89] Levin M. (2023). Bioelectric networks: The cognitive glue enabling evolutionary scaling from physiology to mind. Anim. Cogn..

[90] Pezzulo G., Levin M. (2016). Top-down models in biology: Explanation and control of complex living systems above the molecular level. J. R. Soc. Interface.

[91] Nanos V., Levin M. (2022). Multi-scale Chimerism: An experimental window on the algorithms of anatomical control. Cells Dev..

[92] Lobo D., Solano M., Bubenik G.A., Levin M. (2014). A linear-encoding model explains the variability of the target morphology in regeneration. J. R. Soc. Interface.

[93] Levin M. (2019). The Computational Boundary of a “Self”: Developmental Bioelectricity Drives Multicellularity and Scale-Free Cognition. Front. Psychol..

[94] Levin M. (2022). Technological Approach to Mind Everywhere: An Experimentally-Grounded Framework for Understanding Diverse Bodies and Minds. Front. Syst. Neurosci..

[95] Pio-Lopez L., Bischof J., LaPalme J.V., Levin M. (2023). The scaling of goals from cellular to anatomical homeostasis: An evolutionary simulation, experiment and analysis. Interface Focus.

[96] Langton C.G. (1997). Artificial Life: An Overview.

[97] Von Neumann J., Burks A.W. (1966). Theory of self-reproducing automata. IEEE Trans. Neural Netw..

[98] Games M. (1970). The fantastic combinations of John Conway’s new solitaire game “life” by Martin Gardner. Sci. Am..

[99] Chan B.W.C. (2019). Lenia: Biology of Artificial Life. Complex Syst..

[100] Li X., Yeh A.G.O. (2002). Neural-network-based cellular automata for simulating multiple land use changes using GIS. Int. J. Geogr. Inf. Sci..

[101] Mordvintsev A., Randazzo E., Niklasson E., Levin M. (2020). Growing Neural Cellular Automata. Distill.

[102] Pontes-Filho S., Walker K., Najarro E., Nichele S., Risi S. A single neural cellular automaton for body-brain co-evolution. Proceedings of the 22nd Annual Conference on Genetic and Evolutionary Computation, ACM, GECCO ’22.

[103] Hansen N., Ostermeier A. (2001). Completely Derandomized Self-Adaptation in Evolution Strategies. Evol. Comput..

[104] Vandenberg L.N., Morrie R.D., Adams D.S. (2011). V-ATPase-dependent ectodermal voltage and ph regionalization are required for craniofacial morphogenesis. Dev. Dyn..

[105] Hamming R.W. (1950). Error detecting and error correcting codes. Bell Syst. Tech. J..

[106] Wang C., Sklar D., Johnson D. (2001). Forward error-correction coding. Crosslink.

[107] Zhang Y., Fontaine M.C., Bhatt V., Nikolaidis S., Li J. (2023). Arbitrarily Scalable Environment Generators via Neural Cellular Automata. arXiv.

[108] Rumelhart D.E., Hinton G.E., Williams R.J. (1986). Learning internal representations by error propagation. Parallel Distributed Processing: Explorations in the Microstructure of Cognition, Volume 1: Foundations.

[109] Hiscock T.W. (2019). Adapting machine-learning algorithms to design gene circuits. BMC Bioinform..

[110] Wolfram S. (2002). A New Kind of Science.

[111] Cook M. (2004). Universality in Elementary Cellular Automata. Complex Syst..

[112] Hornik K., Stinchcombe M., White H. (1989). Multilayer feedforward networks are universal approximators. Neural Netw..

[113] Sutton R.S., Barto A.G. (2018). Reinforcement Learning: An Introduction.

[114] Tang Y., Ha D. (2021). The Sensory Neuron as a Transformer: Permutation-Invariant Neural Networks for Reinforcement Learning. Adv. Neural Inf. Process. Syst..

[115] Wolpert L. (1971). Chapter 6 Positional Information and Pattern Formation. Curr. Top. Dev. Biol..

[116] Sharpe J. (2019). Wolpert’s French Flag: What’s the problem?. Development.

[117] Gabalda-Sagarra M., Carey L.B., Garcia-Ojalvo J. (2018). Recurrence-based information processing in gene regulatory networks. Chaos Interdiscip. J. Nonlinear Sci..

[118] Biswas S., Manicka S., Hoel E., Levin M. (2021). Gene regulatory networks exhibit several kinds of memory: Quantification of memory in biological and random transcriptional networks. iScience.

[119] Biswas S., Clawson W., Levin M. (2022). Learning in Transcriptional Network Models: Computational Discovery of Pathway-Level Memory and Effective Interventions. Int. J. Mol. Sci..

[120] Etcheverry M., Moulin-Frier C., Oudeyer P.Y., Levin M. (2023). AI-Driven Automated Discovery Tools Reveal Diverse Behavioral Competencies of Biological Networks.

[121] Frank S.A. (2023). Robustness and complexity. Cell Syst..

[122] Frank S.A. (2003). Genetic variation of polygenic characters and the evolution of genetic degeneracy. J. Evol. Biol..

[123] Frank S.A. (2004). Genetic variation in cancer predisposition: Mutational decay of a robust genetic control network. Proc. Natl. Acad. Sci. USA.

[124] Frank S.A. (2009). The common patterns of nature. J. Evol. Biol..

[125] Frank S.A. (2013). Evolution of Robustness and Cellular Stochasticity of Gene Expression. PLoS Biol..

[126] Frank S.A. (2018). Measurement invariance explains the universal law of generalization for psychological perception. Proc. Natl. Acad. Sci. USA.

[127] Frank S.A. (2019). Evolutionary design of regulatory control. II. Robust error-correcting feedback increases genetic and phenotypic variability. J. Theor. Biol..

[128] Bongard J., Levin M. (2023). There’s Plenty of Room Right Here: Biological Systems as Evolved, Overloaded, Multi-Scale Machines. Biomimetics.

[129] Gershenson C., Helbing D. (2015). When slower is faster. Complexity.

[130] Mordvintsev A., Randazzo E., Fouts C. (2022). Growing Isotropic Neural Cellular Automata. Artificial Life Conference Proceedings 34.

[131] Sohl-Dickstein J., Weiss E., Maheswaranathan N., Ganguli S. (2015). Deep Unsupervised Learning using Nonequilibrium Thermodynamics. Proc. Mach. Learn. Res..

[132] Ho J., Jain A., Abbeel P. (2020). Denoising diffusion probabilistic models. Adv. Neural Inf. Process. Syst..

[133] Nichol A.Q., Dhariwal P. (2021). Improved Denoising Diffusion Probabilistic Models. Proc. Mach. Learn. Res..

[134] Dhariwal P., Nichol A. (2021). Diffusion Models Beat GANs on Image Synthesis. Adv. Neural Inf. Process. Syst..

[135] Song J., Meng C., Ermon S. (2022). Denoising Diffusion Implicit Models. arXiv.

[136] Savic D., Belintzev B., Beloussov L., Zaraisky A. (1986). Morphogenetic activity prepattern in embryonic epithelia. Prog. Clin. Biol. Res..

[137] Hunt P., Krumlauf R. (1991). Deciphering the Hox code: Clues to patterning branchial regions of the head. Cell.

[138] Ashe H.L., Briscoe J. (2006). The interpretation of morphogen gradients. Development.

[139] Levin M., Martyniuk C.J. (2018). The bioelectric code: An ancient computational medium for dynamic control of growth and form. Biosystems.

[140] Palm R.B., Duque M.G., Sudhakaran S., Risi S. (2022). Variational Neural Cellular Automata. arXiv.

[141] Stanley K.O. (2007). Compositional pattern producing networks: A novel abstraction of development. Genetic Program. Evolvable Mach..

[142] Woolley B.G., Stanley K.O. On the deleterious effects of a priori objectives on evolution and representation. Proceedings of the 13th Annual Conference on Genetic and Evolutionary Computation, ACM, GECCO ’11.

[143] Mildenhall B., Srinivasan P.P., Tancik M., Barron J.T., Ramamoorthi R., Ng R. (2021). NeRF: Representing scenes as neural radiance fields for view synthesis. Commun. ACM.

[144] Kamm R.D., Bashir R. (2013). Creating living cellular machines. Ann. Biomed. Eng..

[145] Teague B.P., Guye P., Weiss R. (2016). Synthetic morphogenesis. Cold Spring Harb. Perspect. Biol..

[146] Parr T., Pezzulo G., Friston K.J. (2022). Active Inference: The Free Energy Principle in Mind, Brain, and Behavior.

[147] Ha D., Schmidhuber J. (2018). Recurrent World Models Facilitate Policy Evolution. Advances in Neural Information Processing Systems.

[148] Pontes-Filho S., Lind P.G., Nichele S. (2022). Assessing the robustness of critical behavior in stochastic cellular automata. Phys. D Nonlinear Phenom..

[149] Beggs J.M. (2008). The criticality hypothesis: How local cortical networks might optimize information processing. Philos. Trans. R. Soc. A Math. Phys. Eng. Sci..

[150] Shew W.L., Plenz D. (2012). The Functional Benefits of Criticality in the Cortex. Neuroscientist.

[151] Shew W.L., Clawson W.P., Pobst J., Karimipanah Y., Wright N.C., Wessel R. (2015). Adaptation to sensory input tunes visual cortex to criticality. Nat. Phys..

[152] Clawson W.P., Wright N.C., Wessel R., Shew W.L. (2017). Adaptation towards scale-free dynamics improves cortical stimulus discrimination at the cost of reduced detection. PLoS Comput. Biol..

[153] Habibollahi F., Kagan B.J., Burkitt A.N., French C. (2023). Critical dynamics arise during structured information presentation within embodied in vitro neuronal networks. Nat. Commun..

[154] Jones S.A., Barfield J.H., Norman V.K., Shew W.L. (2023). Scale-free behavioral dynamics directly linked with scale-free cortical dynamics. eLife.

[155] Wissner-Gross A.D., Freer C.E. (2013). Causal Entropic Forces. Phys. Rev. Lett..

[156] Pio-Lopez L., Levin M. (2024). Aging as a Morphostasis Defect: A Developmental Bioelectricity Perspective. ARR.

[157] Miller W.B., Baluška F., Reber A.S., Slijepčević P. (2024). Why death and aging? All memories are imperfect. Prog. Biophys. Mol. Biol..

[158] McCulloch W.S., Pitts W. (1943). A logical calculus of the ideas immanent in nervous activity. Bull. Math. Biophys..

[159] Minsky M.L. (1954). Theory of Neural-Analog Reinforcement Systems and Its Application to the Brain-Model Problem.

[160] Minsky M. (1961). Steps toward artificial intelligence. Proc. IRE.

[161] Rosenblatt F. (1962). Principles of Neurodynamics: Perceptrons and the Theory of Brain Mechanisms.

[162] Minsky M., Papert S. (1969). Perceptron: An introduction to Computational Geometry.

[163] Rumelhart D.E., Hinton G.E., Williams R.J. (1986). Learning representations by back-propagating errors. Nature.

[164] LeCun Y., Bengio Y., Hinton G. (2015). Deep learning. Nature.

[165] Goodfellow I., Bengio Y., Courville A. (2016). Deep Learning.

[166] Paszke A., Gross S., Massa F., Lerer A., Bradbury J., Chanan G., Killeen T., Lin Z., Gimelshein N., Antiga L. (2019). PyTorch: An Imperative Style, High-Performance Deep Learning Library. Advances in Neural Information Processing Systems 32.

[167] Hansen N., Kadlecová G., Nozawa K., Rolshoven L., Chan M., Akimoto Y., Brockhoff D., yoshihikoueno, ARF1, brieglhostis (2023). CMA-ES/pycma: r3.3.0.

[168] Wagner A. (2012). The role of robustness in phenotypic adaptation and innovation. Proc. R. Soc. B Biol. Sci..

